# Jasmonate-mediated defence responses, unlike salicylate-mediated responses, are involved in the recovery of grapevine from *bois noir* disease

**DOI:** 10.1186/s12870-017-1069-4

**Published:** 2017-07-10

**Authors:** Anna Rita Paolacci, Giulio Catarcione, Luisa Ederli, Claudia Zadra, Stefania Pasqualini, Maurizio Badiani, Rita Musetti, Simonetta Santi, Mario Ciaffi

**Affiliations:** 1 Dipartimento per la Innovazione nei Sistemi Biologici, Agroalimentari e Forestali, Università della Tuscia, Via S. Camillo De Lellis, s.n.c, I-01100 Viterbo, Italy; 2 Dipartimento di Chimica, Biologia e Biotecnologie, Università di Perugia, Borgo XX Giugno, 74, I-06121 Perugia, Italy; 3 Dipartimento di Scienze Farmaceutiche, Università di Perugia, Borgo XX Giugno, 74, I-06121 Perugia, Italy; 4 Dipartimento di Agraria, Università Mediterranea di Reggio Calabria, Loc. Feo di Vito, I-89129 Reggio Calabria, Italy; 5 Dipartimento di Scienze Agroalimentari, Ambientali e Animali, Università di Udine, Via delle Scienze, 206, I-33100 Udine, Italy

**Keywords:** *Bois noir* disease and recovery, Grapevine, Jasmonate, Phytoplasmas, Plant-pathogen interactions, Salicylate, Stolbur, *Vitis vinifera* L

## Abstract

**Background:**

*Bois noir* is an important disease of grapevine (*Vitis vinifera* L.), caused by phytoplasmas. An interesting, yet elusive aspect of the *bois noir* disease is “recovery”, i.e., the spontaneous and unpredictable remission of symptoms and damage. Because conventional pest management is ineffective against *bois noir*, deciphering the molecular bases of recovery is beneficial. The present study aimed to understand whether salicylate- and jasmonate-defence pathways might have a role in the recovery from the b*ois noir* disease of grapevine.

**Results:**

Leaves from healthy, *bois noir*-diseased and *bois noir*-recovered plants were compared, both in the presence (late summer) and absence (late spring) of *bois noir* symptoms on the diseased plants. Analyses of salicylate and jasmonate contents, as well as the expression of genes involved in their biosynthesis, signalling and action, were evaluated. In symptomatic diseased plants (late summer), unlike symptomless plants (late spring), salicylate biosynthesis was increased and salicylate-responsive genes were activated. In contrast, jasmonate biosynthesis and signalling genes were up-regulated both in recovered and diseased plants at all sampling dates. The activation of salicylate signalling in symptomatic plants might have antagonised the jasmonate-mediated defence response by suppressing the expression of jasmonate-responsive genes.

**Conclusions:**

Our results suggest that grapevine reacts to phytoplasma infection through salicylate-mediated signalling, although the resultant full activation of a salicylate-mediated response is apparently ineffective in conferring resistance against *bois noir* disease. Activation of the salicylate signalling pathway that is associated with the presence of *bois noir* phytoplasma seems to antagonise the jasmonate defence response, by failing to activate or suppressing both the expression of some jasmonate responsive genes that act downstream of the jasmonate biosynthetic pathway, as well as the first events of the jasmonate signalling pathway. On the other hand, activation of the entire jasmonate signalling pathway in recovered plants suggests the potential importance of jasmonate-regulated defences in preventing *bois noir* phytoplasma infections and the subsequent development of *bois noir* disease. Thus, on one hand, recovery could be achieved and maintained over time by preventing the activation of defence genes associated with salicylate signalling, and on the other hand, by activating jasmonate signalling and other defence responses.

**Electronic supplementary material:**

The online version of this article (doi:10.1186/s12870-017-1069-4) contains supplementary material, which is available to authorized users.

## Background

Phytoplasmas are mycoplasma-like pathogens that cause serious yield losses worldwide in economically important crops [1]. They are prokaryotes that belong to the Mollicutes class, a group of wall-less micro-organisms that are phylogenetically related to low G + C gram-positive bacteria. Phytoplasmas are obligate parasites of plants and insects that need both hosts for their dispersal in nature. In host plants, they are restricted to the phloem and are transmitted in a persistent manner by phloem sap-feeding leafhoppers or psyllids.

Grapevine yellows represent a group of widespread diseases of grapevine (*Vitis vinifera* L.) that display common symptoms, which are traceable to molecularly distinguishable phytoplasmas. The most important diseases in the main viticultural areas of Europe are *flavescence dorée* and *bois noir* [1]. Even though *bois noir* is not considered a quarantine disease by the European Plant Protection Organization, as *flavescence dorée* is, it nevertheless has considerable impact on viticulture production [2]. Indeed, although *bois noir* is often endemic, severe epidemics can also occur, as has been reported in several Italian regions over the past several years [3].

The *bois noir* disease is associated with phytoplasmas of the stolbur group (16SrXII-A), known as ‘*Candidatus Phytoplasma solani*’, which is transmitted by the polyphagous leafhopper vector *Hyalesthes obsoletus* Signoret (Hemiptera, Cixiidae) [4]. Typical visible symptoms of *bois noir* infection on grapevine do not become evident before late summer in Italy, and include leaf curling; discoloration of leaf veins and laminas, i.e., yellowing or reddening, according to the cultivar; abnormal lignification of the canes; flower abortion; and berry withering. Such symptoms have been related to several plant physiological modifications, including reduced photosynthetic rate, stomatal closure and anomalous accumulation of carbohydrates in leaves, which are the main likely causes of the dramatic reduction in yield, observed after *bois noir* infection [2, 5].

Preventive measures, such as the use of healthy propagating materials and treatments against the vector, do not decrease the incidence of *bois noir*, mainly because insect vectors dwell on herbaceous plants, from which they acquire the stolbur phytoplasma, and only feed occasionally on grapevine [6]. Common pathogen eradication practices, such as roguing of symptomatic plants, are also ineffective, because the infected grapevines are not a direct source of infection [5]. Furthermore, the absence of genetic sources of resistance and the impossibility of culturing phytoplasma in vitro have greatly delayed the development of control methods, even as the biochemical and molecular mechanisms involved in the phytoplasma/plant interaction are becoming clearer [7–11].

An interesting, but still elusive aspect of the phytoplasma-plant interaction is “recovery”, observed in both *bois noir*- and *flavescence dorée*-infected grapevines, i.e., a spontaneous remission of symptoms, during which the causal agent disappears from the crown [5, 10, 11]. Once recovered, grapevines do not acquire permanent immunity from *bois noir*; they may actually be re-infected in the field, but this invariably occurs to a lesser extent [5]. Because there are no effective, direct means of reducing the incidence of *bois noir*, deciphering the molecular bases of recovery has become a major topic of interest, in view of its potential use as a control strategy against this and other phytoplasmas in grapevine.

Previous studies on a range of tree crops, such as apple and grapevine, suggest that recovery from phytoplasma-associated diseases is linked to changes in the oxidative status of the phloem, in which an accumulation of H_2_O_2_, a stable reactive oxygen species (ROS), the antimicrobial and signalling roles of which are well known, invariably occurs [10, 12, 13].

The ROS interacts with a network of signal transduction pathways, in which the phytohormones salicylate and jasmonate act as secondary messengers. Salicylate/jasmonate interplay has a major role in the development of disease symptoms and the hypersensitive response [14], as well as the activation of distinct sets of defence-related genes [15], leading to the establishment of local and/or systemic resistance [16].

As very little is known about the importance of salicylate- and jasmonate-mediated signalling in phytoplasma/plant interactions, the present study aimed to determine whether these defence pathways are activated in response to stolbur phytoplasma infection in grapevine. Thus, to ascertain whether metabolic and molecular changes are associated with the plant response to phytoplasma infection and/or the development of symptoms, the leaves of healthy (H), diseased (D), and recovered (R) plants were sampled for two consecutive years in late summer, when *bois noir* symptoms were apparent on D plants. A third sampling was carried out between the aforementioned sampling intervals, i.e. in the late spring of the second year, when *bois noir* symptoms on D plants were not yet visible. In the leaf samples obtained, the salicylate and jasmonate contents were measured, and expression of the genes involved in their biosynthesis and signalling, as well as the changes in expression of different downstream salicylate- and jasmonate-responsive genes, such as those coding for specific pathogenesis-related proteins (PRP) and WRKY transcription factors (TFs), were analysed. In addition, given the documented importance of flavonoids and stilbenoids in other biotic interactions in grapevine [17, 18], and considering the role of jasmonate and salicylate in the induction of their synthesis [19], the expression of genes coding for key biosynthetic enzymes, such as stilbene and chalcone synthases, was also analysed.

## Results

### Stolbur molecular markers are detectable in symptomatic diseased (S-D) leaves, but not in non-symptomatic diseased (NS-D) leaves

*Bois noir*-diseased (symptomatic, D); healthy (never symptomatic, H); and recovered grapevines (R, i.e., plants symptomatic and found positive for *bois noir* in the past, but *bois noir*-negative and symptomless within the preceding 2 years) were compared. Five plants each, of the D, H and R groups of plants, were randomly selected in the vineyard upon first sampling, in August 2011, and maintained throughout the study. Fully expanded, coeval, intact leaves, collected from each plant at three different time points, specifically in August 2011, June 2012 and August 2012, were analysed.

The D plants showed visible *bois noir* foliar symptoms in August 2011 and 2012 (data not shown). These symptomatic D plants were referred to as S-D. In contrast, *bois noir* symptoms were not apparent in D plants sampled in June 2012 (non-symptomatic D plants, NS-D). During the three sampling periods, H and R plants remained consistently symptomless and visually indistinguishable from each other.

Real-time RT-PCR identified the presence of stolbur marker transcripts in S-D leaves only (both years), and thus, not in NS-D, nor in R or H leaves (data not shown).

### Salicylate is increased in S-D leaves only, and not in NS-D leaves

**Fig. 1:**
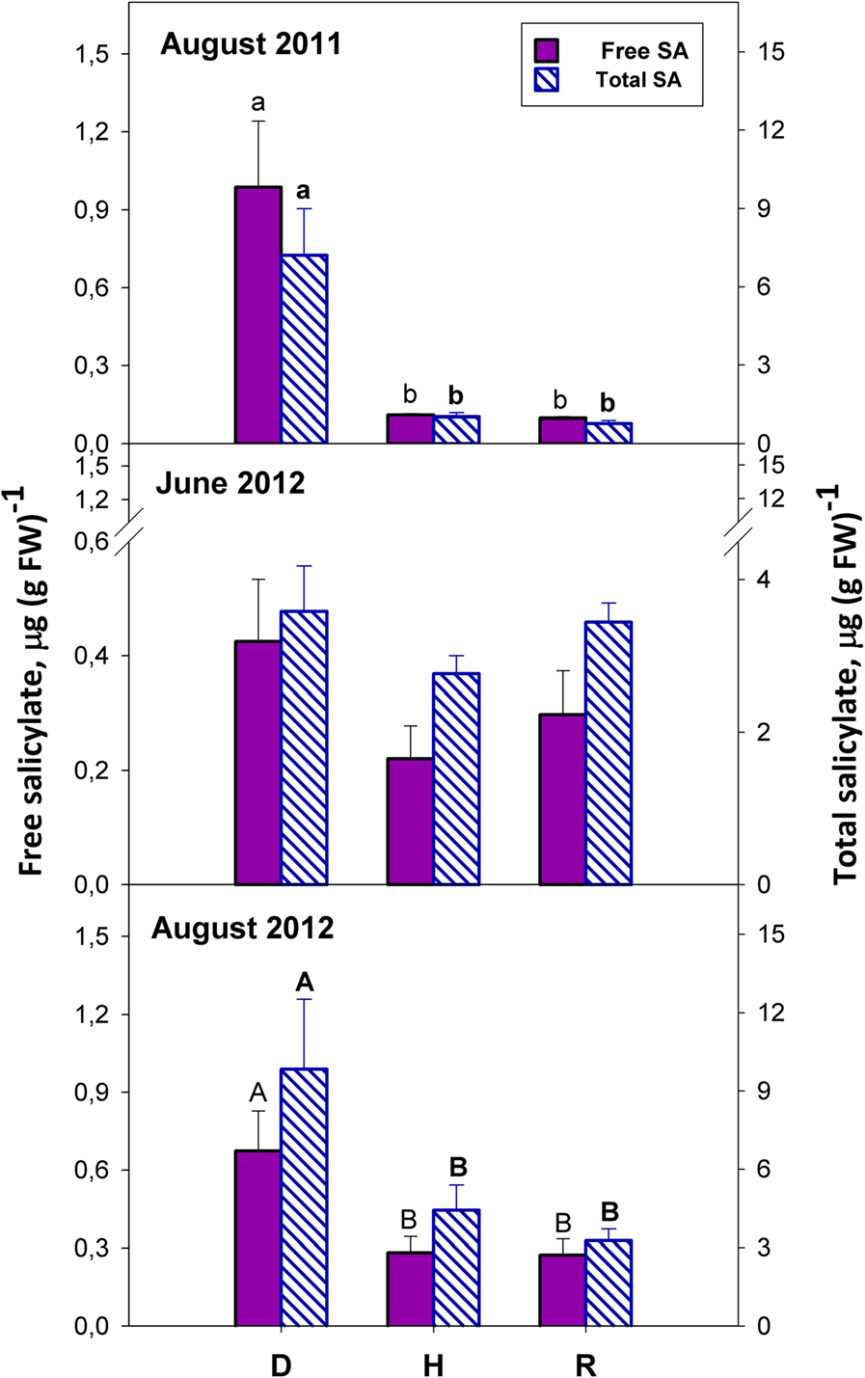
Free and total salicylate (SA) in the leaves of *bois noir*-diseased (D), healthy (H), and recovered (R) Chardonnay grapevines, collected when symptoms were apparent (August 2011 and 2012) or still latent (June 2012). Each value represents the mean ± SE of five biological replicates (*n* = 5). Different letters, when present, denote significant differences at *P* ≤ 0.01 (lowercase or common) or at *P* ≤ 0.05 (uppercase or capital); regular font or bold letters are used for free or total salicylate, respectively

Compared to H and R leaves, free salicylate in S-D ones was about seven-times higher on August 2011 and three-times higher on August 2012 (Fig. 1). In contrast, on June 2012, when the *bois noir* symptoms were still not apparent, no significant difference was detected among NS-D, H and R plants (Fig. 1). Total salicylate, i.e. the sum of the free form and of its glucosidic conjugates, closely matched the distribution of free salycilate among the experimental variants in the different sampling periods (Fig. 1).

### Methyl-jasmonate is increased in S-D, NS-D and R leaves

**Fig. 2:**
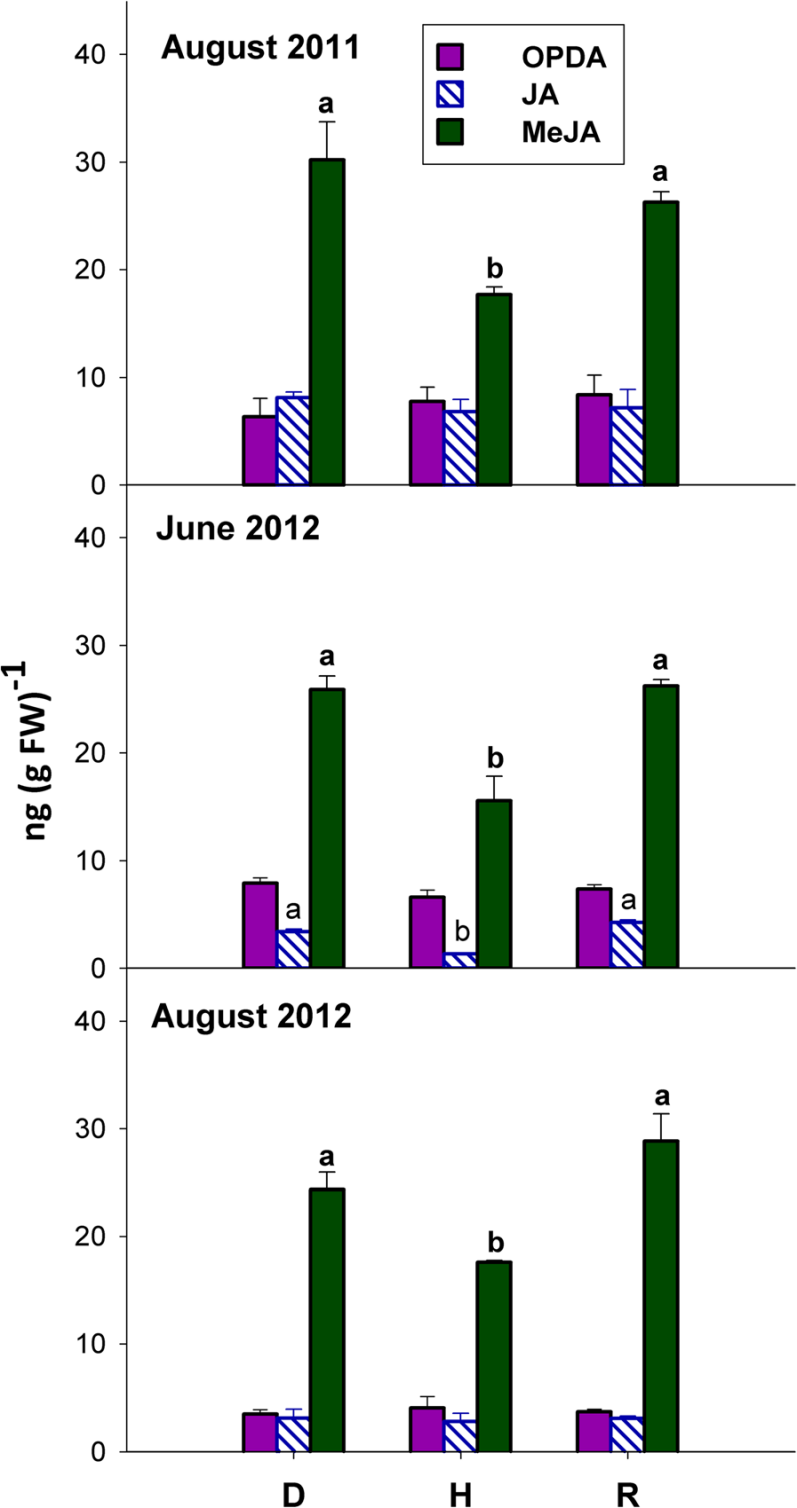
Levels of 12-oxo-phytodienoic acid (OPDA), jasmonate (JA) and methyl-jasmonate (MeJA) in the grapevine leaves of Fig. 1. Statistics as in Fig. 1. Letters denoting significant differences are in regular font or bold font for JA or MeJA, respectively

In the plant material studied here, jasmonate was always found to be much less abundant than its methylated derivative (Fig. 2). In comparison with H leaves, the infection brought about by ‘Ca. *P. solani*’, either previously occurred (R leaves) or extant (D leaves), caused an increase in the levels of methyl-jasmonate, irrespective of the presence of visible *bois noir* symptoms in D leaves. A comparable increase in D and R leaves was also seen for jasmonate, but only on June 2012, i.e., when bois noir symptoms were still not apparent in D plants (Fig. 2). No difference was ever observed among experimental variants in the three sampling periods for the jasmonate precursor 12-oxo-phytodienoic acid (OPDA; Fig. 2).

### Most salicylate biosynthetic genes are up-regulated in D leaves

A database search led to identify 12 functional sequences coding for phenylalanine ammonia-lyase (PAL) and a single isochorismate synthase (ICS) gene in the *V. vinifera* genome, the details of which are presented in Additional file 1: Table S1. Owing to its very low, and in some cases, even null expression levels in the plant material under consideration (data not shown), *VvPAL12* was not considered any further.

**Fig. 3:**
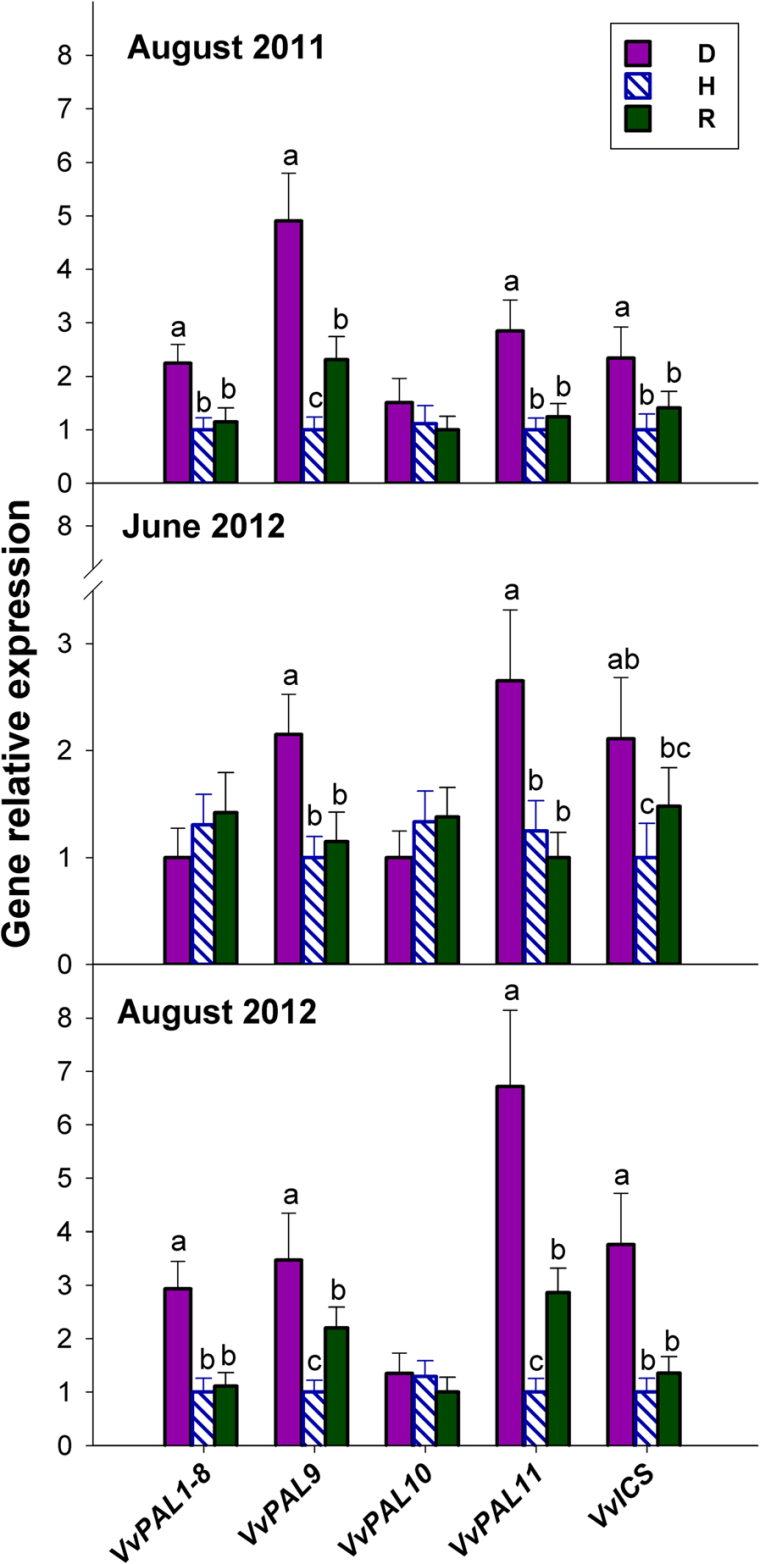
Relative expression levels of genes coding for phenylalanine ammonia-lyase (PAL) and isochorismate synthase (ICS) in the grapevine leaves of Fig. 1. For each gene, relative expression levels were calculated by setting a value of 1, as the lowest value among *bois noir*-diseased (D), healthy (H), or recovered (R) plants. The expression data of each gene were normalised using the geometric average of the two reference genes *VvEF1α* and *VvGAPDH*. Their normalised relative values are presented as the mean ± SD of five biological replicates, each of which was analysed in triplicate (*n* = 15). Statistics as in Fig. 1

In comparison to H leaves, *VvPAL9* and *VvPAL11* were significantly up-regulated in both S-D (both years) and NS-D leaves (Fig. 3); whereas *VvPAL1–8* was up-regulated in S-D plants alone. To a lesser extent, *VvPAL9* and *VvPAL11* were also up-regulated in R leaves, but only in August 2011 and August 2012 (*VvPAL9)* and August 2012 *(VvPAL11).* No differences were observed in the expression of *VvPAL10*. Furthermore, expression of the single *ICS* gene found in the *V. vinifera* genome was up-regulated in S-D and NS-D plants, in comparison to H and R plants (Fig. 3).

### Most jasmonate biosynthetic genes are up-regulated in S-D, NS-D and R leaves

A database search for grapevine genes coding for the major enzymes involved in jasmonate biosynthesis and modification, such as lipoxygenase (LOX), allene oxide synthase (AOS), allene oxide cyclase (AOC), 12-OPDA reductase (OPR) and jasmonate carboxyl methyltransferase (JMT), led to observe that most of the components of the jasmonate biosynthetic pathway in grapevine are encoded by multiple genes that are organised into small gene families (Additional file 1: Table S2).

**Fig. 4:**
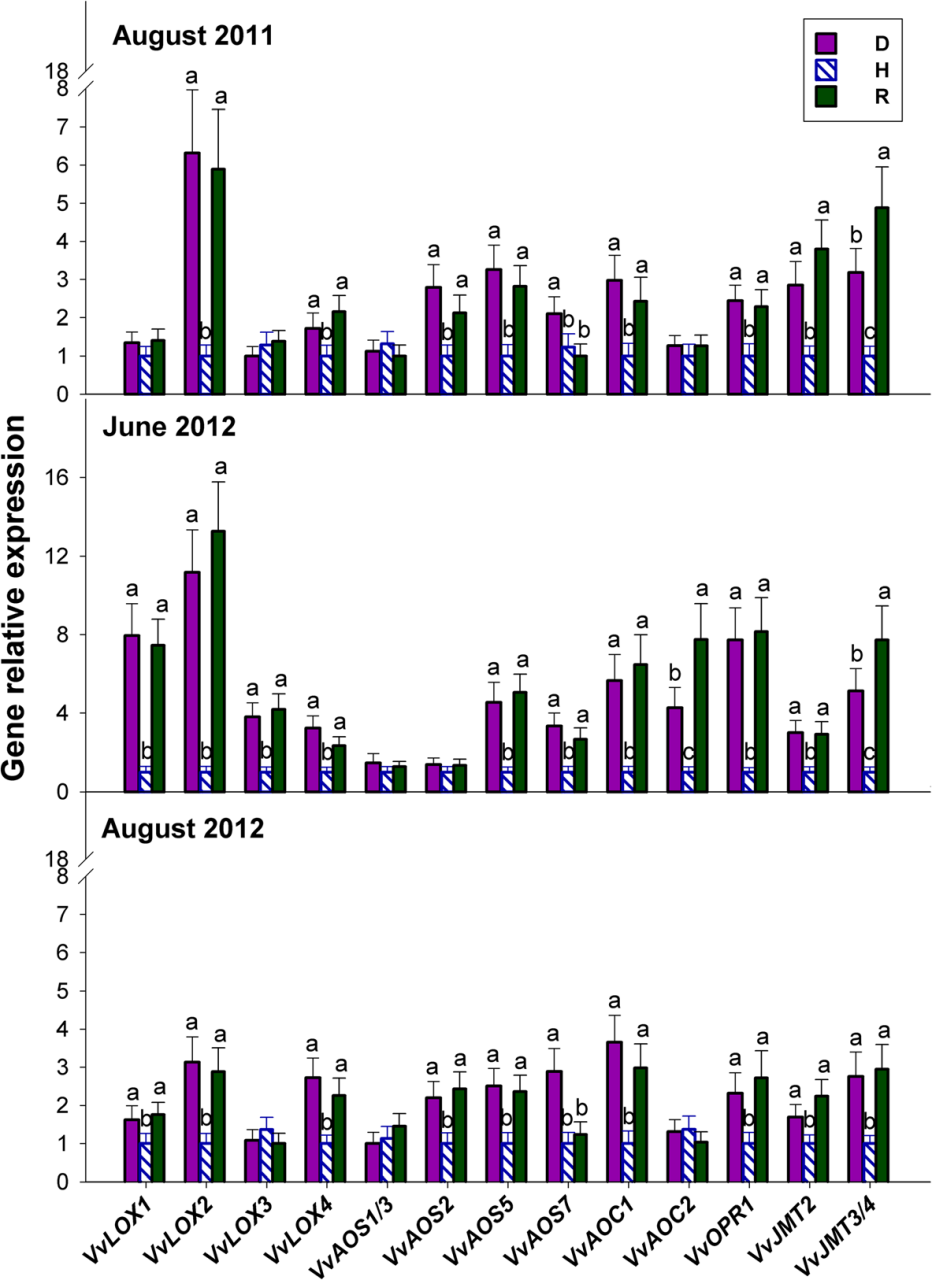
Relative expression levels of genes coding for lipoxygenase (LOX), allene oxide synthase (AOS), allene oxide cyclase (AOC), OPDA reductase (OPR) and jasmonate carboxyl methyltransferase (JMT) in the grapevine leaves of Fig. 1. Calibration, normalisation, sample replication and statistics as in Fig. 3

Four *LOX* genes were identified in the *V. vinifera* genome, the details of which are given in Additional file 1: Table S2. In comparison to H leaves, the relative expression of *VvLOX2* and *VvLOX4*, particularly the former, was significantly higher in S-D (for both years), NS-D and R plants; whereas that of *VvLOX1* and *VvLOX3* was significantly increased only in NS-D and R leaves (Fig. 4). Seven functional *AOS* genes were identified in the *V. vinifera* genome (Additional file 1: Table S2); however, because of its very low or even null transcripts abundance in the plant material used in the present study, the gene pair *VvAOS4/6* was not considered any further. The genes *VvAOS2* and *VvAOS5* showed very similar expression patterns in August 2011 and August 2012, with increased expression in S-D and R plants in comparison to H plants; whereas in June 2012, only *VvAOS5* was up-regulated in NS-D and R leaves (Fig. 4). In August of both years, *VvAOS7* expression was higher in S-D plants than in H and R plants; whereas in June 2012, its behaviour was similar to that of *VvAOS5* (Fig. 4). Moreover, the expression of *VvAOS1/3* showed no change among experimental variants and sampling dates (Fig. 4).

In June 2012, the two *AOC* genes identified in the *V. vinifera* genome were both up-regulated in NS-D, and particularly in R leaves, in comparison to H leaves (Fig. 4). The transcription levels of *VvAOC1*, but not those of *VvAOC2*, also showed a similar pattern in the leaves collected in August of both years (Fig. 4).

A database search led to identify 11 distinct *V. vinifera* genes coding for OPR, of which only one, namely *VvOPR1,* showed a high level of sequence similarity with the *OPR3-*like genes specifically involved in jasmonate biosynthesis [20]. Therefore, specific primers for expression analysis were designed only for *VvOPR1*. Similar to *VvAOC1*, significant up-regulation of *VvOPR1* was observed in R, S-D and NS-D leaves, especially evident in June 2012 (Fig. 4).

Of the four genes coding for JMT, identified in the *V. vinifera* genome, *VvJMT1* was not any considered further, because the level of its transcripts was very low or even null in the plant material. Similar to most of the genes involved in jasmonate biosynthesis, *VvJMT2* and *VvJMT3/4* were both up-regulated in S-D, NS-D and R plants, in comparison to H plants (Fig. 4). It is worth noting that in August 2011 and June 2012, the relative expression of *VvJMT3/4* was at its lowest in H plants, intermediate in D plants and significantly higher in R plants (Fig. 4).

### Salicylate signalling genes are up-regulated in S-D leaves alone, whereas jasmonate signalling genes are up-regulated in S-D, NS-D and R leaves

Three genes involved in the salicylate signalling pathway, namely the *nonexpressor of pathogenesis-related* (PR) genes 1 (*VvNPR1.1* and *VvNPR1.2*) and the *enhanced disease susceptibility* (*VvEDS1*) gene, in addition to four genes involved in the jasmonate signalling pathway that have been previously characterised in grapevine, namely the *myelocytomatosis* (*VvMYC2*) and *jasmonate ZIM-domain genes* (*VvJAZ1*, *VvJAZ2* and *VvJAZ3*), were evaluated in the present study (Additional file 1: Table S3).

**Fig. 5:**
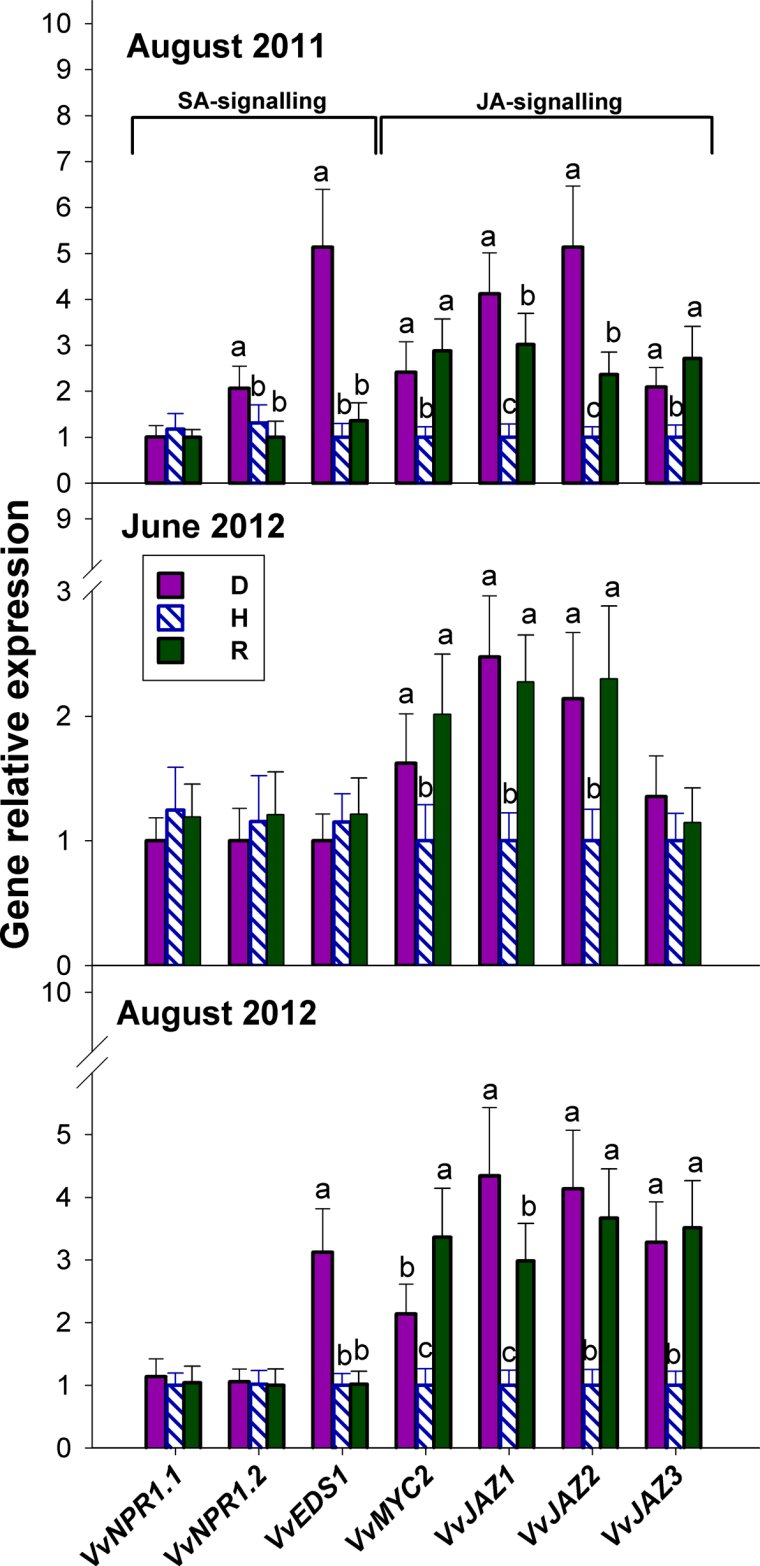
Relative expression levels of genes coding for components of the salicylate (SA) or jasmonate (JA) signalling pathways, namely nonexpressor of PR1 (NPR1), enhanced disease susceptibility (EDS1), myelocytomatosis (MYC2) and jasmonate ZIM-domain (JAZ), in the grapevine leaves of Fig. 1. Calibration, normalisation, sample replication and statistics as in Fig. 3

No differences were observed in the relative expression of the *VvNPR1* genes among D, H and R plants, with the exception of only a slight up-regulation of *VvNPR1.2* in S-D leaves in August 2011 (Fig. 5). In contrast, the other salicylate signalling gene under consideration, namely *VvEDS1*, was strongly up-regulated in S-D plants, in comparison to H and R plants, but showed no up-regulation in NS-D plants (Fig. 5). As has been observed for most genes involved in jasmonate biosynthesis, all *V. vinifera* genes coding for putative components of the jasmonate signalling pathway were significantly up-regulated in S-D, NS-D and R plants, in comparison to H plants. The only exception was *VvJAZ3*, the expression of which was unaffected within the three groups of plants during June 2012 (Fig. 5).

### Salicylate-responsive and jasmonate-responsive WRKY genes are regulated in S-D leaves in an opposing fashion, whereas *VvWRKY2* is selectively up-regulated in R leaves

**Fig. 6:**
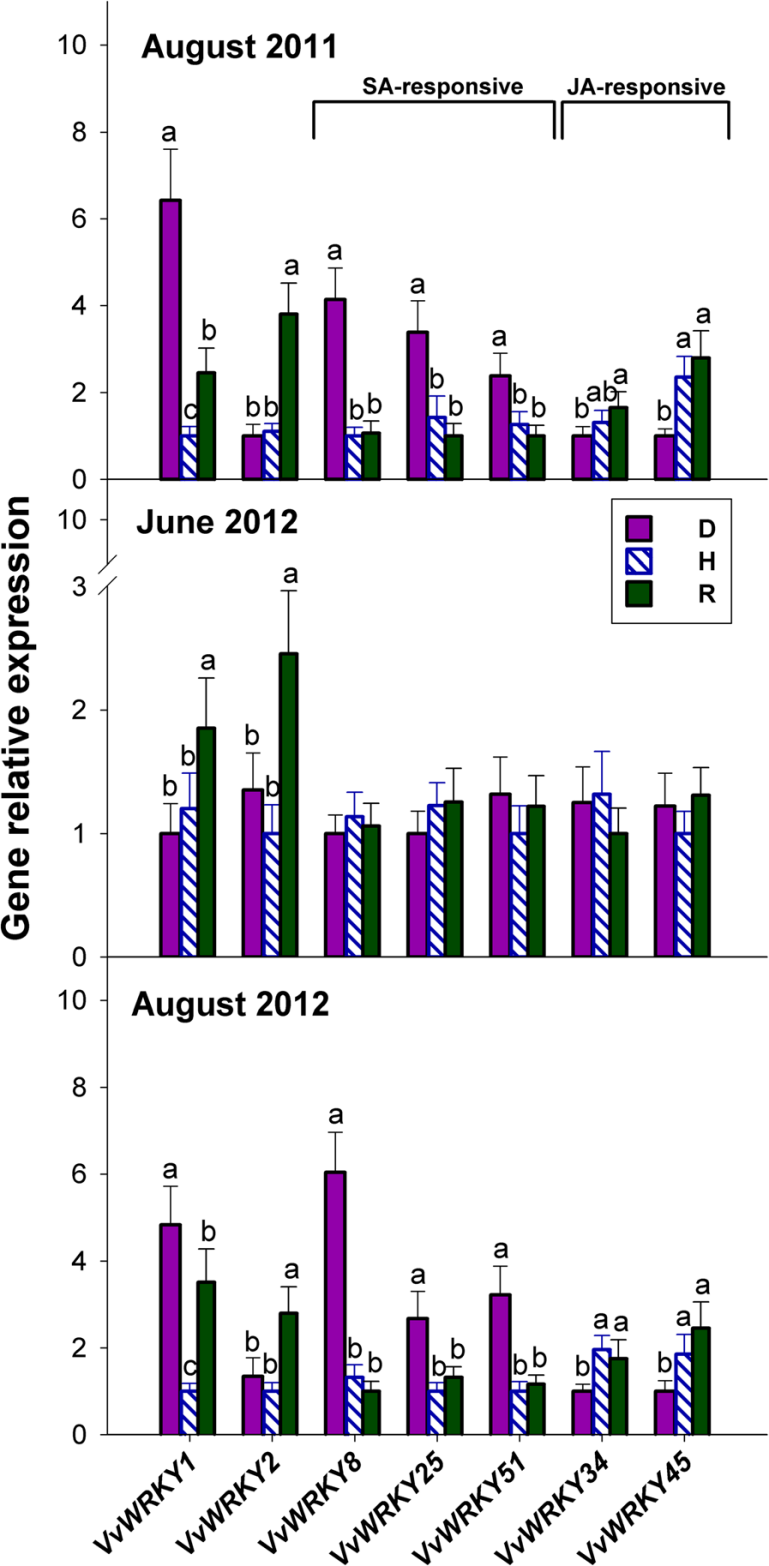
Relative expression levels of genes coding for WRKY transcription factors in the grapevine leaves of Fig. 1. Responsiveness to salicylate (SA) or jasmonate (JA), when known, is indicated. Calibration, normalisation, sample replication and statistics as in Fig. 3

In the present study, seven representative members of the grape WRKY TFs gene family were selected and analysed (details in Additional file 1: Table S4). The *VvWRKY1* gene was significantly up-regulated in S-D plants, compared to H plants, but showed no up-regulation in NS-D plants; whereas it was consistently up-regulated in R plants. In contrast, *VvWRKY2* was consistently up-regulated in R plants alone, in comparison to D and H plants (Fig. 6). Regarding the other five *VvWRKY* genes, no significant differences were observed among D, H and R plants in June 2012. In contrast, much greater diversity in expression levels was apparent in August 2011 and 2012, although individual genes generally tended to maintain the same pattern from year to year (Fig. 6). The transcript levels of the three salicylate-responsive *WRKY* genes (namely *VvWRKY8*, *VvWRKY25* and *VvWRKY51*) were significantly higher in S-D plants than in R and H plants. In contrast, expression of the two jasmonate-inducible *WRKY* genes (namely *VvWRKY34* and *VvWRKY45*) was slightly, yet significantly, down-regulated in S-D plants in comparison to R and H plants.

### Salicylate-responsive genes coding for pathogenesis-related proteins (PRP) are all up-regulated in S-D leaves, whereas most jasmonate-responsive genes are up-regulated in R leaves

A literature search led to identify 15 genes coding for PRP that are potentially involved in the defence response of *V. vinifera,* the details of which are presented in Additional file 1: Table S5.

**Fig. 7:**
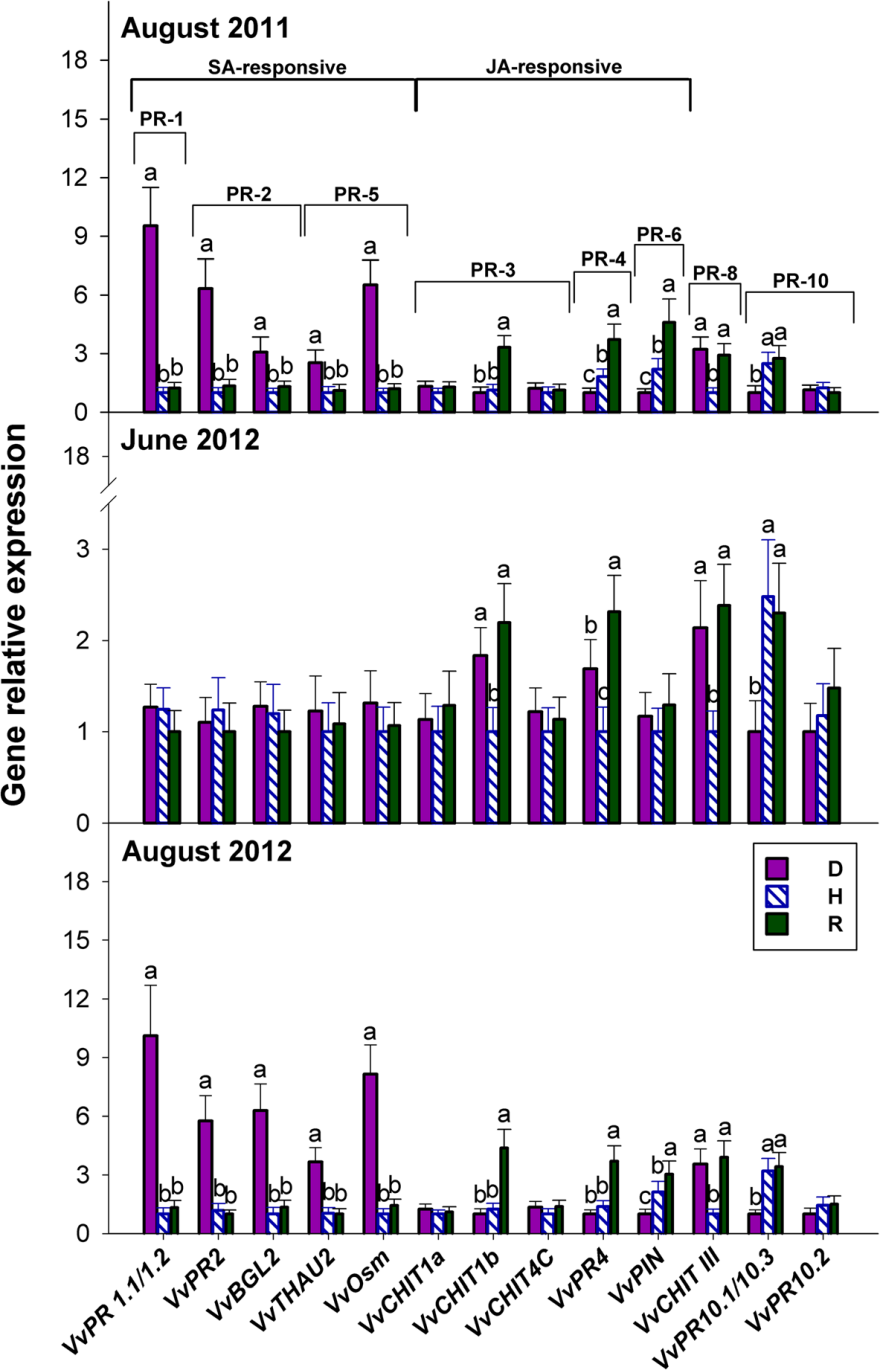
Relative expression levels of genes coding for pathogenesis-related (PR) proteins in the grapevine leaves of Fig. 1. PR gene families and their responsiveness to salicylate (SA) or jasmonate (JA), when known, are indicated. Calibration, normalisation, sample replication and statistics as in Fig. 3

Figure 7 shows that in comparison to H plants, *PRP* expression patterns can be categorised as follows:

1. genes up-regulated in S-D plants alone (both years), namely *VvPR1.1/1.2*, *VvPR2*, *VvBGL2*, *VvTHAU2* and *VvOsm*;
2. genes up-regulated in R plants at all sampling dates, and also in NS-D plants, such as *VvCHIT1b* and *VvPR4;*
3. genes up-regulated in S-D, NS-D and R plants, such as *VvCHITIII*;
4. genes down-regulated in S-D plants, but up-regulated in R plants, such as *VvPIN*;
5. genes down-regulated in S-D and NS-D plants, such as *VvPR10.1/10.3*;
6. genes showing no differences among D, H and R plants at all sampling dates; these included *VvCHIT1a* and *VvCHIT4C*, as well as *VvPR10.2*.

Furthermore, it is again worth noting that the expression patterns of each of the PRP genes under investigation were remarkably similar among each other in August 2011 and August 2012 (Fig. 7).

### Genes coding for stilbene synthase (STS) are up-regulated in S-D leaves, and a subset of these genes are up-regulated in R leaves

A database search led to identify 31 functional *STS* genes (details in Additional file 1: Table S6); however, owing to their very low or null expression in the plant material under investigation (data not shown), *VvSTS13*, *VvSTS19*, *VvSTS31,* and *VvSTS9–11* genes were not considered any further.

**Fig. 8:**
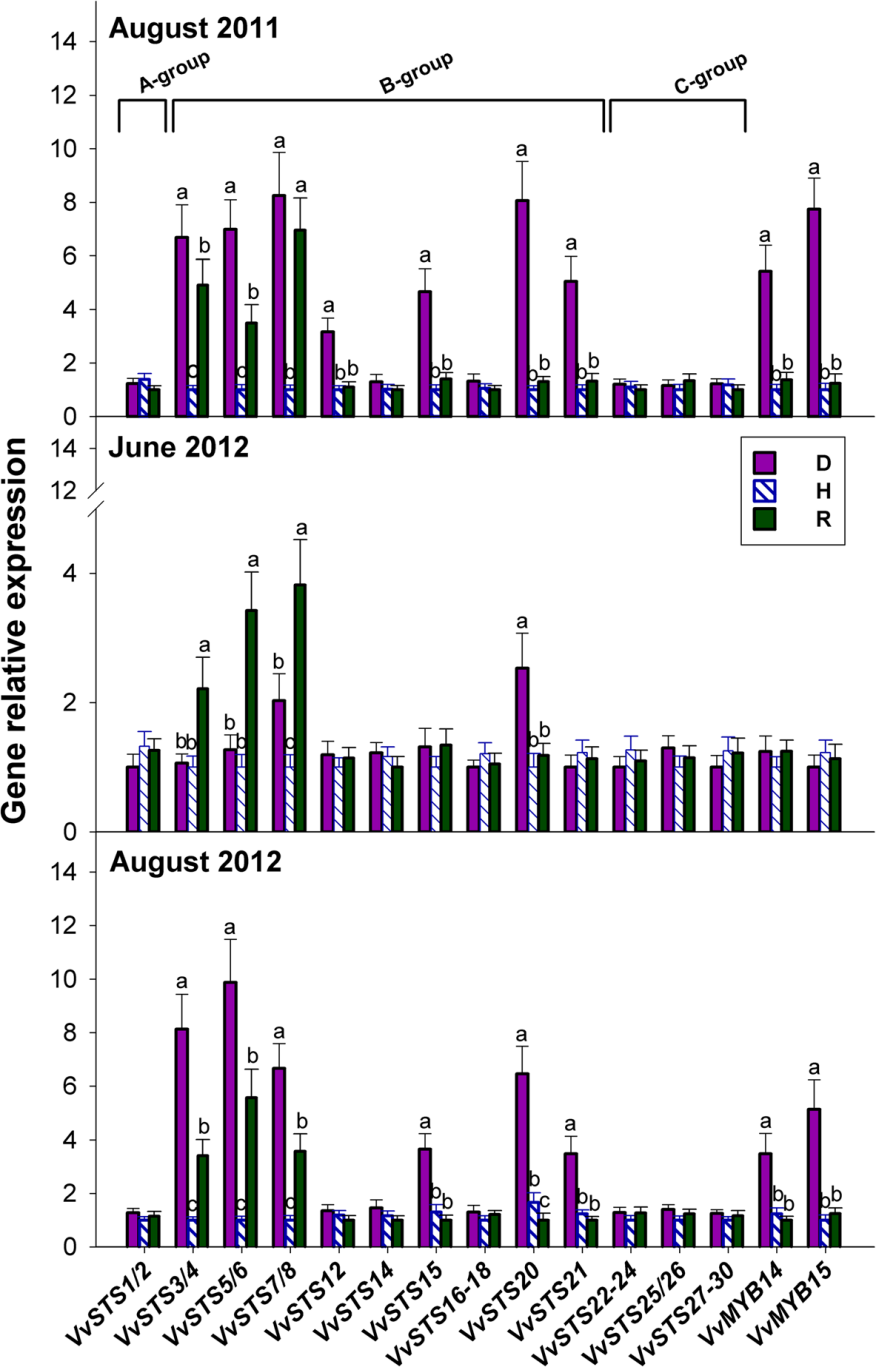
Relative expression levels of genes coding for stilbene synthase (STS) and MYB transcription factors in the grapevine leaves of Fig. 1. The STS phylogenetic groups are indicated. Calibration, normalisation, sample replication and statistics as in Fig. 3

Figure 8 shows that the expression of *VvSTS1/2*, *VvSTS14*, *VvSTS16–18, VvSTS22–24*, *VvSTS25/26* and *VvSTS27–30* genes showed no variations among D, H and R plants*.* The expression of the remaining *STS* genes was instead modulated with respect to the controls (H), to variable extents as follows: *VvSTS3/4, VvSTS5/6, VvSTS15* and *VvSTS21* were up-regulated in S-D plants alone, and remarkably, *VvSTS7/8* and *VvSTS20* were up-regulated in both S-D and NS-D plants; *VvSTS12* was moderately up-regulated only in the 2011 S-D plants. Among the aforementioned genes, the three pairs of very similar *STS* sequences, namely *VvSTS3/4, VvSTS5/6* and *VvSTS7/8* were significantly and stably up-regulated in R leaves (Fig. 8).

Two members of the grape *MYB* gene family of transcription factors, namely *VvMYB14* and *VvMYB15,* were also included in the present study, because they code for TFs that are specifically able to activate the promoter of *STS* genes [21] (Additional file 1: Table S6). These two *MYB* genes were strongly up-regulated in S-D plants alone (Fig. 8).

### Most chalcone synthase genes are up-regulated in S-D leaves, and one such gene is up-regulated in NS-D leaves

A database search allowed the identification of five functional chalcone synthase (*CHS*) genes (Additional file 1: Table S7). Owing to its very low or null expression in the plant material under investigation (data not shown), the *VvCHS5* gene was not considered any further.

**Fig. 9:**
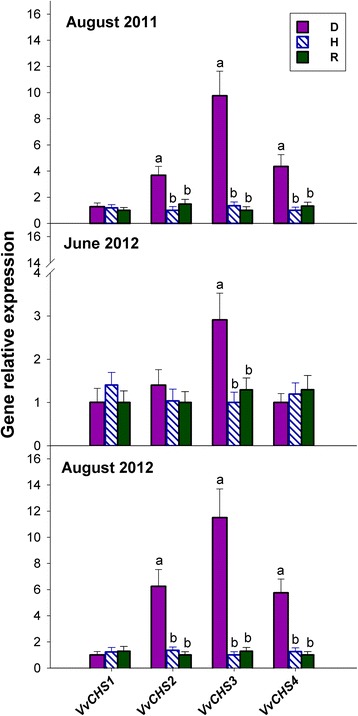
Relative expression levels of genes coding for calchone synthase in the grapevine leaves of Fig. 1. Calibration, normalisation, sample replication and statistics as in Fig. 3

Figure 9 shows that with the exception of *VvCHS1*, all *CHS* genes were up-regulated in S-D plants, in comparison to H and R plants. In one case, namely *VvCHS3*, up-regulation was also evident in NS-D plants*.*

## Discussion

### "Ca. *P. solani*" molecular markers are undetectable in the crown of both *bois noir*-recovered and diseased plants early in the vegetative season, before visible symptoms develop

In the present study, the presence of ‘Ca. *P. solani*’ molecular markers in leaf tissues was associated with the presence of disease symptoms, as the stolbur marker transcripts were only detectable in symptomatic leaves, but not in symptomless leaves. This is consistent with the findings of Landi and Romanazzi [9], who detected the *bois noir* phytoplasma in symptomatic leaves of infected grapevines of the cv. Chardonnay and Sangiovese, but not in symptomless leaves of the same plants collected at different phenological stages, according to the presence or absence of *bois noir* symptoms. It is also confirmed here that R grapevines do not host phytoplasmas in their canopies [11, 13]; thus, epidemiologically speaking, they are indistinguishable from healthy, never-infected plants.

### Enhanced salicylate biosynthesis and signalling is involved in the development of *bois noir* symptoms, but not in the induction and maintenance of recovery

The results presented in Fig. 1 indicate that salicylate could be a diagnostic marker for the *bois noir* disease and/or for the development of *bois noir* symptoms, but also show that it might not be involved in the induction and maintenance of recovery. The D grapevines consistently exhibited permanently enhanced expression of certain *PAL* genes and the single *V. vinifera ICS* gene (Fig. 1), both of which are key components of the two alternative pathways that lead to salicylate biosynthesis [22]. The latter is the preferential route during plant/pathogen interactions, specifically in *Arabidopsis* [23]. Apart from salicylate biosynthesis, PAL catalyses the first committed step in the phenylpropanoid pathway, leading to the synthesis of plant defence compounds, such as phytoalexins and lignin monomers [24]. As leaf symptoms in phytoplasma-infected plants have been associated with an anomalous accumulation of carbohydrates [9], their consumption via incorporation into phenylpropanoids has been proposed [25]. Moreover, alterations in polyphenol contents, as well as that of other secondary metabolites associated with increased PAL activity, has been previously reported in grapevine during phytoplasma infection [26], and is indirectly supported at the transcriptional level [7, 8]. These findings are suggestive of the involvement of polyphenols in the response against phytoplasmas. Moreover, the up-regulation of *VvPAL9* and *VvPAL11* in R plants (Fig. 1) also suggests the possible involvement of the phenylpropanoid pathway in recovery.

As far as components of the salicylate signalling pathway are concerned, the results obtained for *VvNPR1.1* and *VvNPR1.2* (Fig. 5) confirm the previous findings of Le Henanff et al. [27] in their study on the *Vitis*/*Plasmopara viticola* pathosystem, and suggest, according to Dong [28], that *NPR1* expression could be post-transcriptionally regulated in grapevine. In contrast, the expression of another salicylate signalling gene, namely *VvEDS1*, closely matched the patterns of salicylate accumulation in S-D leaves (compare Fig. 1 with Fig. 5). In *Arabidopsis*, *EDS1*, a key regulator of basal resistance to host-adapted biotrophic pathogens, has been implicated in a salicylate-dependent positive feedback loop, which up-regulates defence responses in host cells that are in immediate contact with the pathogen and surrounding cells [29]. Consistent with the present results, it has been previously reported that expression of *VvEDS1* is stimulated either after inoculation with *Botrytis cinerea* or *P. viticola*, or by salicylate treatment [30], thus confirming that *VvEDS1* could play an important role in salicylate-mediated signalling responses to pathogens.

### Activation of the oxylipin pathway and its downstream signalling components is an early, subliminal indicator of *bois noir* infection, which is also involved in the establishment/maintenance of recovery

Jasmonates were originally associated with defence against herbivores and necrotrophic pathogens [31], but they have also been more recently implicated in resistance against biotrophs, such as powdery and downy mildews, in *Arabidopsis* and grapevine [32–35]. Therefore, the accumulation of methyl-jasmonate and jasmonate observed in D plants of the present study (Fig. 2) might be a defence response directed against *bois noir* infection, or the insect vector *H. obsoletus*, or both. However, the salicylate accumulation observed in those same plants (Fig. 1) suggests that a possible salicylate/jasmonate interplay could ultimately determine the outcome of *bois noir* disease.

By comparing Fig. 2 with Fig. 1, it can be deduced that unlike salicylate, jasmonate accumulation occurred both in NS-D and R plants. On one hand, this might suggest that jasmonate accumulation might be regarded as an early, subliminal indicator of *bois noir* infection, and on the other hand, it suggests that the oxylipin pathway, and possibly its downstream signalling components (see below), might have a role in the establishment and/or maintenance of recovery.

Figure 2 shows that methyl-jasmonate was significantly more abundant and responsive to the grapevine/phytoplasma interaction than jasmonate was. Indeed, recent evidence reinforces the idea that methyl-jasmonate, because of its volatile nature, is more effective than jasmonate, as a transmissible signal that is able to induce systemic defence responses in plants [36]. Therefore, the increased methyl-jasmonate levels detected in both D and R plants could reflect a systemic jasmonate response following grapevine/phytoplasma interaction.

Mirroring the pattern of jasmonate accumulation, the expression of several genes involved in jasmonate biosynthesis was always significantly up-regulated in both D and R plants, particularly in June 2012 (Fig. 4), suggesting enhanced activity of the oxylipin pathway in these plants during an early stage of their growing season. Mimicking the promotive action of exogenous jasmonate application on the expression of several genes involved in jasmonate biosynthesis [37], the increased levels of endogenous jasmonates observed in the present study, following grapevine/phytoplasma interaction, could activate a positive feedback loop leading to an amplified jasmonate response.

The 13-LOX enzymes catalyse the initial step of jasmonate formation in plants [37], and expression of the corresponding genes rapidly increases in response to abiotic and biotic stress, such as wounding or pathogens. In *V. vinifera*, expression of the *13-LOX* gene *VvLOXO,* previously identified in the berries of the cv. Sauvignon Blanc, and corresponding to the most responsive *LOX* gene considered in the present study (*VvLOX2*), matched the distribution of jasmonate within different berry tissues, and was found to be selectively induced in response to wounding or *B. cinerea* infection [38]. Although not directly involved in jasmonate biosynthesis, 9-LOXs have also been proposed to play an important role in local and systemic defence responses against pathogens, such as programmed cell death and the production of antimicrobial compounds [39]. Expression of the previously identified *VvLOXC* gene, corresponding to *VvLOX4* in the present study, was strongly increased in the cv. Chardonnay [30] and Sauvignon Blanc [38], infected with *P. viticola* and *B. cinerea*, respectively. Moreover, expression of a putative *9-LOX* gene was considerably increased after jasmonate treatment in seedling leaves of the cv. Chassellas [40]. Taken together, the above results support the view that both the *13*- and *9*-*LOX* genes under investigation in the present study might be involved in the defence response to phytoplasma infection, and possibly in the recovery process.

Recently, Jang et al. [36] reported that the expression of an *Arabidopsis JMT* gene is rapidly induced after jasmonate treatment. This suggests that plants rapidly synthesise methyl-jasmonate from jasmonate to activate the jasmonate response, and that such expression is localised in the phloem, thus affecting systemic jasmonate response to wounding. Conceivably, increased production of methyl-jasmonate in D and R plants (Fig. 2), likely supported by enhanced expression of the *JMT* genes in these same plants (Fig. 4), could be involved in the activation of a systemic jasmonate response in grapevine/phytoplasma interactions.

In *Arabidopsis*, the JAZ/TYFY proteins are considered the main repressors of jasmonate responses [41], whereas MYC2 is the main factor that triggers the jasmonate response, by direct interaction with the JAZ proteins [42]. In the same species, jasmonate biosynthesis and signalling are interlinked by a positive feedback loop, whereby jasmonates stimulate the expression of both jasmonate biosynthetic genes, and those genes involved in its signalling, including those coding for the JAZ/TYFY repressor proteins [43]. The results reported here for *VvJAZ1–3* and *VvMYC2* (Fig. 5) support the foregoing viewpoint, and suggest an enhanced sensitivity of the jasmonate signalling pathway, probably in response to increased jasmonate levels in D and R plants. Consistently, expression of the same four jasmonate signalling genes in the present study was strongly induced in *V. vinifera* cell cultures, in response to exogenous jasmonate treatment [44, 45]. This suggests that a positive feedback loop regulatory mechanism linking jasmonate biosynthesis and signalling might also be conserved in grapevine.

### *VvWRKY2*, known to play a role in the regulation of lignin biosynthesis, is involved in the recovery from *bois noir* via jasmonate signalling

Most WRKY TFs activate the expression of defence genes in response to biotic and abiotic stress through salicylate- and/or jasmonate-dependent pathways [46]. Here, the relative expression of three *WRKY* genes previously known to be strongly induced upon exogenous salicylate treatment, namely *VvWRKY8*, *VvWRKY25* and *VvWRKY51* [47], mirrored the salicylate prevalence in diseased plants, particularly in S-D plants (compare Fig. 6 and Fig. 1). Because *bois noir* infection has been found to effect strong induction of these same *WRKY* genes in the susceptible cv. Chardonnay, but much less in the tolerant cv. Incrocio Manzoni [7, 47], this suggests that their up-regulation could indicate an established phytoplasma infection, rather than the deployment of an effective defence response. In contrast, the results shown in Fig. 6 for two jasmonate-inducible *WRKY* genes, namely *VvWRKY34* and *VvWRK45* [45], could suggest that their expression is negatively regulated by activation of the salicylate signalling pathway in D plants. Such hypothesis could be supported by the results obtained by Albertazzi et al. [7] and by Wang et al. [47] who found that *bois noir* infection decreases the expression of *VvWRKY45* in the susceptible cv Chardonnay, but slightly induces it in the tolerant cv Incrocio Manzoni. Another one of the *WRKY* genes studied here, namely *VvWRKY1,* was previously found to be induced by salicylate [48]. Accordingly, Fig. 6 shows that *VvWRKY1* was up-regulated in S-D plants, i.e., coinciding with salicylate accumulation in these same plants (Fig. 1). Marchive et al. [49] reported that *VvWRKY1* over-expression in grapevine leads to increased tolerance to *P. viticola*, probably via jasmonate-mediated transcriptional reprogramming. Indeed, we report here that this gene was consistently up-regulated in R plants (Fig. 6), which accumulated jasmonate, but not salicylate (see Figs. 1 and 2). Taken together, the above results suggest that *VvWRKY1* is involved in defence responses mediated by both salicylate and jasmonate, independently of any possible crosstalk between the two.

Figure 6 shows that in contrast to *VvWRKY1*, *VvWRKY2* was consistently up-regulated in R plants alone, in comparison to both H and D plants. This supports the findings of Gambino et al. [10], who worked on a different grapevine cv., namely Barbera, affected by the *flavescence dorée* disease. Furthermore, Mzid et al. [50] showed that unlike *VvWRKY1* (see above), *VvWRKY2* is not induced by salicylate in grapevine leaves; neither does its over-expression in tobacco cause activation of salicylate-inducible *PR* genes, but rather enhances tolerance to necrotrophic fungi through the activation of genes probably involved in jasmonate-dependent responses. Therefore, the up-regulation of *VvWRKY2* observed in R plants suggests that the establishment/maintenance of the condition of recovery could be connected to jasmonate signalling, but is independent of salicylate signalling. Moreover, Guillaumie et al. [51] showed that *VvWRKY2* might play a role in regulating lignin biosynthesis, suggesting that its over-expression in grapevine could limit the colonisation of vineyards by the insects that act as vectors of the *bois noir* and *flavescence dorée* diseases. If this does indeed occur, *VvWRKY2* up-regulation in R grapevines could decrease their susceptibility to re-infection, which is a trait of agronomic interest [5].

### Expression of the jasmonate-responsive PRP genes *VvCHIT1b*, *VvPR4* and *VvPIN* is up-regulated during latent *bois noir* infection, and in recovered plants, but becomes repressed upon the development of symptoms

In *V. vinifera*, the exogenous application of salicylate modulates the expression of *PRP* in the PR-1, PR-2 and PR-5 families; whereas exogenous jasmonate induces mainly *PR-3* (basic chitinase), *PR-4* and *PR-6* genes [30, 34, 40]. Here, confirming once more that salicylate biosynthesis and signalling is involved in the development of *bois noir* symptoms (see above), salicylate-responsive PRPs were all up-regulated in S-D plants alone (Fig. 7). This is consistent with previous observations in *bois noir*-infected [7–9], as well as *flavescence dorée*-infected [10] grapevines. The up-regulation of the two β-1-3-glucanase genes, *VvPR2* and *VvBGL2*, in S-D plants might cause callose degradation, thereby facilitating the spread of phytoplasmas through the phloem [9, 10]. Conversely, the lack of an increase in the expression of glucanases in R plants might cause pathogen-confining callose deposition in the sieve elements, which indeed appears to be a recurring feature during recovery from phytoplasma diseases [8, 52].

In contrast to salicylate-responsive genes, the expression of jasmonate-responsive *PRP* genes was either not modulated (*VvCHIT1b* and *VvPR4*; PR-3 and PR-4 families, respectively) or repressed (*VvPIN*; PR-6) in S-D plants, in comparison to control plants, but was consistently up-regulated in R plants (Fig. 7). Two of the aforementioned jasmonate-responsive *PRP* genes, namely *VvCHIT1b* and *VvPR4*, were also up-regulated in NS-D plants (Fig. 7), indicating the accumulation of jasmonate, but not salicylate (Figs. 1 and 2). Furthermore, *VvCHITIII* was the only *PRP* gene that was consistently up-regulated in both D and R plants (Fig. 7). Coherently with previous findings [9], this could suggest that class III chitinase might play a role both during *bois noir* infection and during recovery.

### “Diseased” or “recovery” status in the stolbur/grapevine interaction depends on the outcome of the salicylate/jasmonate interplay - is an antagonist of jasmonate signalling/action waiting to be discovered?

The results reported here for PRPs and WRKY TFs are consistent with the well-established antagonism between salicylate and jasmonate in mediating defence responses following plant-pathogen interactions. They suggest that salicylate accumulation under extant phytoplasma infection might antagonise the jasmonate defence response, by either failing to activate or suppressing the expression of jasmonate-responsive genes. Such antagonism would occur downstream of the jasmonate biosynthesis and signalling, as suggested both by the present results and by recent literature [53, 54].

The importance of jasmonate-regulated defences in contrasting the *bois noir* disease is suggested by the fact that the entire jasmonate signalling pathway is activated in *bois noir*-recovered plants. Although no direct evidence has been produced so far, that the jasmonate signalling pathway is required for defence against *bois noir* disease, indirect support might come from the observation that partial uprooting and pulling, mimicking mechanical stress and wounding, might induce recovery in *bois noir*- and *flavescence dorée*-infected grapevines [55]. In addition, mounting of an effective jasmonate defence response against *bois noir* phytoplasmas could increase, as a beneficial side effect, grapevine tolerance towards the attack of insect vectors. This could explain why, in cases of re-infection of recovered plants in the field, the severity of disease is invariably lower than it is in original infections [55].

Whether *bois noir* phytoplasmas prevent the mounting of jasmonate defences via salicylate/jasmonate crosstalk, and/or by the production of inhibitors/effectors that antagonise jasmonate signalling or action, remains to be answered. A model supporting the latter hypothesis is the Aster yellows witches’ broom (AY-WB) phytoplasma that is known to interfere with the jasmonate-dependent response via the effector protein, SAP11, and is able to destabilise TFs acting as positive regulators of the *LOX2* gene in *Arabidopsis* [56]. *Arabidopsis* plants that transgenically express SAP11, as well as wild type plants infected with AY-WB, show down-regulated *LOX* expression and reduced jasmonate levels upon wounding. The leafhopper vector *Macrosteles quadrilineatus* has been consistently found to produce more progeny on AY-WB-infected, SAP11-expressing or *LOX2*-silenced plants [57]. However, to date, no analogue of the SAP11 effector has been found in the stolbur phytoplasma.

### Salicylate and jasmonate might activate distinct sets of *STS* genes, and stilbene phytoalexins could be involved in both symptom development and recovery from *bois noir*

Stilbenes, which represent the major class of phytoalexins, and flavonoids, which use chalcone as a biosynthetic precursor, are well-known defence compounds. The levels of these compounds are promptly modulated in many plant species, including grapevine, in response to either biotic or abiotic stress, or to experimental treatments with stress hormones, such as salicylate, jasmonate and ethylene [18, 19, 35]. The STS and CHS enzymes catalyse key reactions in biosynthetic pathways, to produce stilbenes and flavonoids, respectively. Furthermore, they are closely related enzymes that compete for the same substrates; thus, it is not surprising that their transcriptional responses are observed in opposition to each other under certain circumstances [18].

To our knowledge, the present study is the first to report on the comprehensive expression analysis of grapevine *STS* and *CHS* genes, and investigate the potential involvement of stilbenes and flavonoids in the defence response against the *bois noir* phytoplasma. The expression profiles presented in Fig. 8, in which most of the *STS* genes in group B (but not those in groups A and C) were modulated in D, H and R plants, support the previous findings of Vannozzi et al. [18], who showed that the group B grapevine *STS* genes are the most responsive to several biotic and abiotic stresses. Three genes within the subset of modulated group B *STS* genes, namely *VvSTS12*, *VvSTS15* and *VvSTS21*, were found to be up-regulated in S-D plants alone. Their expression patterns among D, H and R plants were closely matched by those of the two TFs *VvMYB14* and *VvMYB15,* which regulate STS genes in grapevine, in response to biotic and abiotic stresses [21] (Fig. 8). Other members of group B, namely the three pairs of very similar genes *VvSTS3/4, VvSTS5/6* and *VvSTS7/8*, were induced not only in S-D grapevines, but also permanently induced in R grapevines (Fig. 8). Thus, we suggest that stilbenes could be associated with both the manifestation of symptoms, as well as the spontaneous remission of symptoms, which reasonably implies a differential regulation of *STS* genes under two divergent circumstances.

Unlike certain members of the *STS* family, modulated *CHS* genes were up-regulated exclusively in D plants (Fig. 9). Among these, *VvCHS3* appeared to be the most responsive to phytoplasma infection, and the only one that was also up-regulated in NS-D leaves. *VvCHS3* was also found to be the most responsive among the *CHS* genes in anthocyanin-accumulating grapevine leaves, infected with the GRLRaV-3 virus [58]. In summary, the induction of *CHS* genes observed in the present study, and the subsequent likely accumulation of flavonoids, could be associated with *bois noir* infection, and/or the development of symptoms, without participation in the phenomenon of recovery.

## Conclusions

The present study reports a detailed molecular characterisation of the jasmonate- and salicylate-mediated defence pathways in response to *bois noir* infection in grapevine. In the pathosystem under investigation, consideration must be given to the fact that functional analysis aimed at deciphering the mechanisms of disease/recovery is considerably limited by a number of constraints as follows: in vitro culture of the pathogen has proven to be extremely difficult; experimental inoculation is invariably futile; techniques for the induction of recovery under controlled conditions, to the best of our knowledge, have not been successful so far; and genetic sources of resistance are not available. To make things even more complex, it has to be considered that *bois noir* disease results from the interaction of three components, i.e., the plant, the pathogen and the insect vector. As a consequence, molecular mechanisms underpinning *bois noir* disease and recovery can only be studied in the field, where standard tools of functional analysis, such as a pharmacological approach, would be subjected to the inconsistencies and interference of field conditions.

**Fig. 10:**
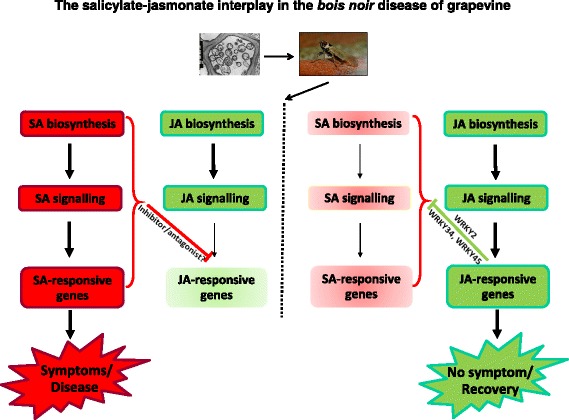
Simplified model showing the salicylate-jasmonate interplay in the *bois noir* disease of grapevine. Plants react to phytoplasma infection by salicylate-mediated signalling, failing to activate or antagonising the jasmonate-mediated defence response, which leads to the development of *bois noir* symptoms and disease (*left panel*). On the other hand, activation of the entire jasmonate signalling pathway, together with counteraction of salicylate signalling and action, inhibits the development of symptoms and phytoplasma disease, leading to recovery from *bois noir* (*right panel*). Acknowledgements: the TEM image showing phytoplasmas infecting a phloem cell is publicly available at: http://dna-barcoding.blogspot.it/2012/12/phytoplasma.html. The picture showing the insect vector Hyalesthes obsoletus is publicly available at: https://www.naturamediterraneo.com/forum/topic.asp?TOPIC_ID=119017

The reported data suggest that grapevine reacts to phytoplasma infection through salicylate-mediated signalling, even though the resultant full activation of a salicylate-mediated response does not appear to be effective in inducing resistance against the *bois noir* disease. Rather, activation of the salicylate signalling pathway that is associated with the presence of *bois noir* phytoplasma, seems to antagonise the jasmonate-mediated defence response, by either failing to activate or suppressing the expression of some jasmonate-responsive genes that act downstream of jasmonate biosynthesis, as well as the first events of the jasmonate signalling pathway (Fig. 10, left panel). On the other hand, activation of the entire jasmonate signalling pathway in recovered plants suggests the potential importance of jasmonate-regulated defences in preventing *bois noir* phytoplasma infections, and the subsequent development of *bois noir* disease. Thus, recovery could be achieved and maintained over time by preventing the activation of defence genes linked to salicylate signalling on one hand, and by activating jasmonate signalling and other defence responses, including increased expression of *WRKY2* and specific *PRP* and *STS* genes on the other hand (Fig. 10, right panel). The involvement of stilbenes in recovery could be particularly meaningful, as they constitute a major class of phytoalexins in grapevine.

## Methods

### Plant material

The present study was carried out in a 0.25 Ha cv. Chardonnay vineyard located near Gorizia (Northeastern Italy). The vineyard had been monitored since 2006 for phytoplasma symptoms and infection. Therefore, a reliable field map of *bois noir*-diseased (symptomatic, D), healthy (never symptomatic, H) and recovered plants (i.e., plants symptomatic and found positive for *bois noir* in the past, but *bois noir*-negative and symptomless over the preceding 2 years, R) was available from the beginning of the present study.

Five plants each, of the D, H, and R groups were randomly selected in the vineyard upon first sampling (August 2011), and maintained throughout the study. Ten fully expanded, coeval, intact leaves were collected from each plant at three different time points: August 2011, June 2012 and August 2012. After excision, the leaves were immediately frozen in liquid nitrogen, and then stored at −80 °C until further analysis.

### *Bois noir* phytoplasma detection

The molecular detection of ‘Ca. *P. solani*’ in the leaves collected from D, H and R plants was performed as described by Santi et al. [59]. Total RNA was extracted from frozen H, D and R leaves using RNeasy® Plant Mini Kit (Qiagen GmbH, Hilden, Germany), and its quantity and purity were evaluated using a NanoDrop ND-1000 UV-Vis Spectrophotometer (Thermo Fisher Scientific, Inc. MA, USA). The RNAs were reverse-transcribed using a QuantiTect® Reverse Transcription Kit (Qiagen GmbH, Hilden, Germany), following the manufacturer’s instructions. Real-Time PCR reactions were set up with the SsoFast EvaGreen® Supermix (Bio-Rad Laboratories Co., Hercules, CA, USA), using specific primers designed on both the 16S rRNA gene of ‘Ca. *Phytoplasma solani’* (accession no. AF248959) and the *DNAK* gene (accession no. AJ970678.1) [59]. Real-Time PCR analyses were performed in a CFX96 Real-Time PCR Detection System (Bio-Rad Laboratories Co., Hercules, CA, USA), using the following standard thermal profile: 95 °C for 3 min; followed by 45 cycles for 5 s at 95 °C; and 5 s at 60 °C. A melting curve analysis of the products was performed from 65 °C to 95 °C to check primer specificity.

### Analysis of salicylate and jasmonate

Total and free salicylate were both extracted and quantified using high-performance liquid chromatography [60]. Jasmonate, as well as its precursor, 12-OPDA, and its gaseous derivative, methyl-jasmonate, were analysed via gas chromatography–mass spectrometry [61].

### Database analyses and identification of target and reference genes

The putative sequences of the genes of interest were retrieved from the National Center for Biotechnology Information (NCBI) database (Additional file 1: Tables S1–S7). Genes responsible for the biosynthesis of salicylate and jasmonate were identified by a BLAST search of the NCBI database, using the available *Arabidopsis* gene sequences [22, 62] (Additional file 1: Tables S1 and S2). Genes coding for STS and CHS were identified, by using the names ‘stilbene synthase’ and ‘chalcone synthase’ as search terms in the *V. vinifera* genome view page (Additional file 1: Tables S6 and S7). Previously isolated and characterised *V. vinifera* sequences were used to retrieve genes that are involved in: salicylate and jasmonate signalling (Additional file 1: Table S3); the defence response to pathogens (PRP and WRKY TFs) (Additional file 1: Tables S4 and S5); and regulation of the stilbene biosynthetic pathway (MYB TFs) (Additional file 1: Tables S6). For each identified gene, the corresponding NCBI mRNA and protein RefSeq sequences were retrieved. The authenticity of the sequences identified was verified by analysing the corresponding RefSeq protein sequences in the Conserved Domains Database (CDD), Pfam hidden Markov models (HMMs), Interpro, and Simple Modular Architecture Research Tool (SMART) databases; whereas the RefSeq mRNA sequences were used as templates to design specific primers for the expression analyses (Additional file 1: Tables S1–S7).

A set of candidate genes showing stable expression in different grape tissues under diverse stress conditions were initially selected to identify the most suitable reference genes (Additional file 1: Table S8). Seven candidate reference genes encoded the following proteins: 60S ribosomal protein L18 (*Vv60SRP*); Actin7 (*VvACT7*); V-type proton ATPase 16 kDa proteolipid subunit (*VvVATP16*); ubiquinol-cytochrome *c* reductase complex chaperone (*VvUQCC*); SAND protein family (*VvSAND*); glyceraldehyde-3-phosphate dehydrogenase (*VvGAPDH*); and EF1-α elongation factor (*VvEF1α*) (Additional file 1: Table S8). Each corresponding mRNA RefSeq was used as the template to design specific primers for the analysis of gene expression.

### RNA extraction, cDNA preparation and gene expression analysis by qRT-PCR

Total RNA was extracted from leaf midribs, following the method described by Gambino et al. [63], with some modifications. Leaf samples (250 mg) were ground in a mortar with liquid nitrogen, and immediately transferred to a micro-centrifuge tube containing 900 μL of pre-warmed (65 °C) extraction buffer (2% cetyl trimethylammonium bromide [CTAB]; 2.5% PVP-40; 2 M NaCl; 100 mM Tris-HCl, pH 8.0; 25 mM EDTA, pH 8.0; and 2% β-mercaptoethanol), vortexed for 2 min and incubated for 10 min at 65 °C. An equal volume of chloroform:isoamyl alcohol (24:1 *v*/v) was added, and the tube was inverted vigorously and centrifuged at 11,000 *g* for 10 min at 4 °C. The supernatant was recovered and a second extraction with chloroform:isoamyl alcohol was performed. The supernatant was then transferred to a new micro-centrifuge tube, and LiCl (3 M final concentration) was added to the mixture, which was left overnight at 4 °C. The RNA was precipitated by centrifugation at 21,000 *g* for 30 min at 4 °C. The pellet was re-suspended in 500 μL of SSTE buffer (1 M NaCl; 1% SDS; 10 mM Tris-HCl, pH 8.0; 1 mM EDTA, pH 8.0) and pre-heated at 65 °C. An equal volume of chloroform:isoamyl alcohol was added and the mixture was centrifuged at 11,000 *g* for 10 min at 4 °C. The supernatant was transferred to a new micro-centrifuge tube and the RNA was precipitated with 0.7 volumes of cold isopropanol, and immediately centrifuged at 21,000 *g* for 15 min at 4 °C. The pellet was washed with ethanol (70%), dried and re-suspended in 100 μL of 0.1% diethyl pyrocarbonate (DEPC)-treated sterile water.

The RNA samples were treated with RNase-free DNase I (Promega, Madison, WI, USA), according to the manufacturer’s protocol. Following digestion, nucleotides were removed from RNA using a G50 Sepharose buffer exchange column (Amersham, Pittsburgh, PA, USA). The RNA concentration and integrity were checked, using a NanoDrop ND-1000 spectrophotometer (Labtech, East Sussex, UK). Only RNA samples with a 260/280 ratio (an index of protein contamination) between 1.9 and 2.1, and a 260/230 ratio (an index of reagent contamination) greater than 2.0, were used for cDNA synthesis. The quality of RNA samples was also assessed by electrophoresis on 1% formaldehyde agarose gels.

First-strand cDNA was synthesised from 3 μg of total RNA using Expand Reverse Transcriptase (Roche Diagnostics, Milano, Italy), according to the manufacturer’s protocol, and the resulting cDNA was diluted fivefold for qRT-PCR analyses.

Specific primer pairs were designed both for the target and selected reference genes (Additional file 1: Tables S1–S8), using the Beacon Designer 6 software (Stratagene, La Jolla, CA), and the following stringency criteria: T_m_ of 55 °C ± 2 °C; PCR amplicon length between 60 and 280 bp; primer length of 21 ± 3 nt; and 40% to 60% guanine-cytosine content. Primers were also designed at the region of the 3′ end of each sequence to encompass all potential splice variants and ensure equal RT efficiencies.

Quantitative RT-PCR analyses were performed using the Mx3000PTM real-time PCR system, with the Brilliant SYBR green QPCR master mix (Stratagene), according to the manufacturer’s protocols, in 25 μL reaction volumes containing 1 μL of each fivefold diluted cDNA, and 150 nM forward and reverse primers. No template nor RT-minus controls were run to detect contamination, dimer formation or the presence of genomic DNA. Standard curves based on five-points, corresponding to a fivefold dilution series (1:1–1:625) from pooled cDNA, were used to compute the PCR efficiency of each primer pair. The PCR efficiency (E) was derived by the eq. E = (10^[−1/m]^ - 1) × 100 [64], where m is the slope of the linear regression model fitted over log-transformed data of the input cDNA concentration versus Ct values, according to the linear equation y = m × log(x) + b. The thermal profile comprised three segments: (i) 95 °C for 10 min; (ii) 40 cycles of 30 s denaturation at 95 °C, 1 min annealing at 55 °C and 30 s extension at 72 °C (amplification data collected at the end of each extension step); and (iii) the dissociation curve, consisting of 1 min incubation at 95 °C, 30 s incubation at 55 °C and a ramp up to 95 °C. Five biological replicates, resulting from five different RNA extractions, and RT and qRT-PCR reactions from five separate plants of each experimental group (D, H and R) at the three different time points under consideration, were used in the quantification analysis. Three technical replicates were analysed for each biological replicate.

Raw C_t_ values were transformed to relative quantities, using the delta-C_t_ formula Q = E^ΔCt^, where E is the efficiency of the primer pair used in the amplification of a particular gene, and ΔC_t_ is the difference between the sample with the lowest C_t_ (highest expression) from the dataset and the C_t_ value of the sample in question.

The expression stability of the seven candidate reference genes (Additional file 1: Table S8) was evaluated, using the software program NormFinder (a Microsoft Excel Add-in available on the Internet), according to the author’s recommendations [65]. The best combination of any two genes recommended by NormFinder was that of *VvEF1α* and *VvGAPDH*, with a stability value significantly lower than that of the most stable gene (*VvEF1α*) considered alone. This indicated a more reliable normalisation than that based on the single most stable gene. Therefore, the expression data of the genes of interest were normalised using the geometric average of the two reference genes *VvEF1α* and *VvGAPDH*, and their normalised relative values were presented as the mean +/−SD. The SDs of normalised expression levels were computed according to the geNorm user manual (geNorm manual, updated 8 July 2008). Expression levels were calibrated by setting a value of 1, as the lowest value among all D, H and R values for each gene, and calculating the remaining two values, accordingly.

### Statistics

Each reported value for the metabolites and gene expression levels represents the mean of five biological replicates, obtained from five individual plants from each experimental group (D, H and R) at the three different time points under consideration. Three technical replicates were analysed for each biological replicate. The statistical significance of the differences observed was evaluated by one-way ANOVA, followed by the Tukey’s test.

## Additional file

Additional file 1: Table S1.
*Vitis vinifera* gene sequences coding for phenylalanine ammonia-lyase (PAL) and isochorismate synthase (ICS), identified in the National Center for Biotechnology Information (NCBI) database and used in the synthesis of oligonucleotides for expression analysis. **Table S2.** *Vitis vinifera* gene sequences coding for lipoxygenase (LOX), allene oxide synthase (AOS), allene oxide cyclase (AOC), 12-oxo-phytodienoic acid (OPDA) reductase (OPR), and jasmonate carboxyl methyltransferase (JMT), identified in the NCBI database and used in the synthesis of oligonucleotides for expression analysis. **Table S3.** *Vitis vinifera* gene sequences involved in salicylate (*NPR1.1*, *NPR1.2* and *EDS1*) or jasmonate (*MYC2*, *JAZ1*, *JAZ2* and *JAZ3*) signalling pathways, identified in the NCBI database and used in the synthesis of oligonucleotides for expression analysis. **Table S4.** *Vitis vinifera* gene sequences coding for WRKY transcription factors, identified in the NCBI database and used in the synthesis of oligonucleotides for expression analysis. **Table S5.** *Vitis vinifera* gene sequences coding for pathogenesis-related proteins (PRP), identified in the NCBI database and used in the synthesis of oligonucleotides for expression analysis. **Table S6.** *Vitis vinifera* gene sequences coding for stilbene synthase (STS) and MYB transcription factors that specifically interact with the *STS* promoter, identified in the NCBI database and used in the synthesis of oligonucleotides for expression analysis. **Table S7.** *Vitis vinifera* gene sequences coding for chalcone synthase (CHS), identified in the NCBI database and used in the synthesis of oligonucleotides for expression analysis. **Table S8.** *Vitis vinifera* sequences coding for candidate reference genes, identified in the NCBI database and used in the synthesis of oligonucleotides for the normalisation of expression data in qRT-PCR analyses. (DOC 358 kb)

## Abbreviations

AOC: Allene oxide cyclase
AOS: Allene oxide synthase
CHS: Chalcone synthase
CTAB: Cetyl trimethylammonium bromide
DEPC: Diethyl pyrocarbonate
JMT: Jasmonate carboxyl methyltransferase
LOX: Lipoxygenase
NCBI: National Center for Biotechnology Information
OPDA: Oxo-phytodienoic acid
OPR: 12-OPDA reductase
PAL: Phenylalanine ammonia-lyase
PRP: Pathogenesis-related proteins
ROS: Reactive oxygen species
STS: Stilbene synthase
TF: Transcription factor
