# Correction to: Jasmonate-mediated defence responses, unlike salicylate-mediated responses, are involved in the recovery of grapevine from *bois noir* disease

**DOI:** 10.1186/s12870-018-1251-3

**Published:** 2018-02-21

**Authors:** Anna Rita Paolacci, Giulio Catarcione, Luisa Ederli, Claudia Zadra, Stefania Pasqualini, Maurizio Badiani, Rita Musetti, Simonetta Santi, Mario Ciaffi

**Affiliations:** 1 Dipartimento per la Innovazione nei Sistemi Biologici, Agroalimentari e Forestali, Università della Tuscia, Via S. Camillo De Lellis, s.n.c, I-01100 Viterbo, Italy; 2 Dipartimento di Chimica, Biologia e Biotecnologie, Università di Perugia, Borgo XX Giugno, 74, I-06121 Perugia, Italy; 3 Dipartimento di Scienze Farmaceutiche, Università di Perugia, Borgo XX Giugno, 74, I-06121 Perugia, Italy; 4 Dipartimento di Agraria, Università Mediterranea di Reggio Calabria, Loc. Feo di Vito, I-89129 Reggio Calabria, Italy; 5 Dipartimento di Scienze Agroalimentari, Ambientali e Animali, Università di Udine, Via delle Scienze, 206, I-33100 Udine, Italy

## Correction

Following publication of the original article [1], it came to the attention of the authors that they had omitted to acknowledge the University of Parma. The Acknowledgement section should read as follows: “The authors kindly acknowledge the University of Parma (Department of Chemistry, Life Sciences and Environmental Sustainability; formerly Department of Life Sciences/Evolutionary and Functional Biology) for the transfer of funds obtained from the Ager project: GIALLUMI DELLA VITE: TECNOLOGIE INNOVATIVE PER LA DIAGNOSI E LO STUDIO DELLE INTERAZIONI PIANTA/PATOGENO, BANDO AGER VITICOLTURA DA VINO”.
