# Supplementary material for: Jasmonate-mediated defence responses, unlike salicylate-mediated responses, are involved in the recovery of grapevine from bois noir disease
Source: BMC Plant Biol. 2017 Jul 10;17:118. doi: 10.1186/s12870-017-1069-4 (PMC5504844; doi:10.1186/s12870-017-1069-4)
Supplement: Additional file 1: Table S1. — Vitis vinifera gene sequences coding for phenylalanine ammonia-lyase (PAL) and isochorismate synthase (ICS), identified in the National Center for Biotechnology Information (NCBI) database and used in the synthesis of oligonucleotides for expression analysis. Table S2. Vitis vinifera gene sequences coding for lipoxygenase (LOX), allene oxide synthase (AOS), allene oxide cyclase (AOC), 12-oxo-phytodienoic acid (OPDA) reductase (OPR), and jasmonate carboxyl methyltransferase (JMT), identified in the NCBI database and used in the synthesis of oligonucleotides for expression analysis. Table S3. Vitis vinifera gene sequences involved in salicylate (NPR1.1, NPR1.2 and EDS1) or jasmonate (MYC2, JAZ1, JAZ2 and JAZ3) signalling pathways, identified in the NCBI database and used in the synthesis of oligonucleotides for expression analysis. Table S4. Vitis vinifera gene sequences coding for WRKY transcription factors, identified in the NCBI database and used in the synthesis of oligonucleotides for expression analysis. Table S5. Vitis vinifera gene sequences coding for pathogenesis-related proteins (PRP), identified in the NCBI database and used in the synthesis of oligonucleotides for expression analysis. Table S6. Vitis vinifera gene sequences coding for stilbene synthase (STS) and MYB transcription factors that specifically interact with the STS promoter, identified in the NCBI database and used in the synthesis of oligonucleotides for expression analysis. Table S7. Vitis vinifera gene sequences coding for chalcone synthase (CHS), identified in the NCBI database and used in the synthesis of oligonucleotides for expression analysis. Table S8. Vitis vinifera sequences coding for candidate reference genes, identified in the NCBI database and used in the synthesis of oligonucleotides for the normalisation of expression data in qRT-PCR analyses. (DOC 358 kb) [file 12870_2017_1069_MOESM1_ESM.doc]

**Manuscript title:**

**Jasmonate-mediated defence responses, unlike salicylate-mediated responses, are involved in the recovery of grapevine from bois noir disease.**

**Authors:**

Anna Rita Paolacci, Giulio Catarcione, Luisa Ederli, Claudia Zadra, Stefania Pasqualini, Maurizio Badiani, Rita Musetti, Simonetta Santi, Mario Ciaffi

The following Additional files are available for the aforementioned manuscript:

**Additional file 1: Table S1.** *Vitis vinifera* gene sequences coding for phenylalanine ammonia-lyase (PAL) and isochorismate synthase (ICS), identified in the National Center for Biotechnology Information (NCBI) database and used in the synthesis of oligonucleotides for expression analysis.

**Additional file 2: Table S2.** *Vitis vinifera* gene sequences coding for lipoxygenase (LOX), allene oxide synthase (AOS), allene oxide cyclase (AOC), 12-oxo-phytodienoic acid (OPDA) reductase (OPR), and jasmonate carboxyl methyltransferase (JMT), identified in the NCBI database and used in the synthesis of oligonucleotides for expression analysis.

**Additional file 3: Table S3.** *Vitis vinifera* gene sequences involved in salicylate (*NPR1.1*, *NPR1.2* and *EDS1*) or jasmonate (*MYC2*, *JAZ1*, *JAZ2* and *JAZ3*) signalling pathways, identified in the NCBI database and used in the synthesis of oligonucleotides for expression analysis.

**Additional file 4: Table S4.** *Vitis vinifera* gene sequences coding for WRKY transcription factors, identified in the NCBI database and used in the synthesis of oligonucleotides for expression analysis.

**Additional file 5: Table S5.** *Vitis vinifera* gene sequences coding for pathogenesis-related proteins (PRP), identified in the NCBI database and used in the synthesis of oligonucleotides for expression analysis.

**Additional file 6: Table S6.** *Vitis vinifera* gene sequences coding for stilbene synthase (STS) and MYB transcription factors that specifically interact with the *STS* promoter, identified in the NCBI database and used in the synthesis of oligonucleotides for expression analysis.

Additional file 7: Table S7. *Vitis vinifera* gene sequences coding for chalcone synthase (CHS), identified in the NCBI database and used in the synthesis of oligonucleotides for expression analysis.

**Additional file 8: Table S8.** *Vitis vinifera* sequences coding for candidate reference genes, identified in the NCBI database and used in the synthesis of oligonucleotides for the normalisation of expression data in qRT-PCR analyses.

Table S1. *Vitis vinifera* gene sequences coding for phenylalanine ammonia-lyase (PAL) and isochorismate synthase (ICS) identified in the National Center for Biotechnology Information (NCBI) database and used for the synthesis of oligonucleotides for expression analysis.

| **Gene** | **NCBI Reference Sequence** | **ORF (bp)** | **Chromosome location** | **Forward primer (5'-3')** | **Reverse primer (5'-3')** |
| --- | --- | --- | --- | --- | --- |
|
|
| *VvPAL1* | XM_002268145 | 2133 | 16 | TGGCAGCACCTCAATCTTCC**^1^** | CCAGCTCCTCCCTCACAAACTT**^1^** |
|
| *VvPAL2* | XM_003633937 | 2133 | 16 | * | * |
|
| *VvPAL3* | XM_002267917 | 2133 | 16 | * | * |
|
| *VvPAL4* | XM_002268220 | 2133 | 16 | * | * |
|
| *VvPAL5* | XM_003633938 | 2133 | 16 | * | * |
|
| *VvPAL6* | XM_002268696 | 2133 | 16 | * | * |
|
| *VvPAL7* | XM_003633939 | 2133 | 16 | * | * |
|
| *VvPAL8* | XM_010663773 | 2133 | 16 | * | * |
|
| *VvPAL9* | XM_002281763 | 2133 | 8 | GGGAACAGGGCTGCTAAC | CGAAAGAAAGTTGGAAGTGG |
|
| *VvPAL10* | XM_010660093 | 2139 | 13 | CCATTTCCAACAGAATCAAG | TCCACTCCTTCAAACAATCC |
|
| *VvPAL11* | XM_002285241 | 2154 | 6 | AAACCGCATCAAGGAGTG | TTTCAGCAAATTGGAAGAGG |
|
| *VvPAL12* | XM_002278480 | 2265 | 11 | CCCAAATCCACCGACTCTG | CACTTCTTTCCTCACAAATC |
|
| *VvICS1* | XM_002267645 | 1683 | 17 | CATCTTGTCTTCTCTTCA | TTGCTTCCTCTAACTATCC |
|

^1^ *VvPAL1-8* form a cluster of genes at the distal end of the chromosome 16 in a region of about 200 kb (580000-790000 bp) and show a high level of similarity among each other in their coding and 5' and 3' untranslated regions (nucleotide identity between 97 and 99%). Due to such remarkable sequence conservation, designing gene-specific oligonucleotidesfor analysing the expression of *VvPAL1-8* genes proved to be very difficult, so that a single conserved pair of primers was designed (denoted with asterisks in the table) to amplify the transcripts of these very similar sequences (referred to as *VvPAL1-8* in the main text). Instead, specific primers were designed for each of the remaining four sequences (*VvPAL9-12*).

**Table S2**. *Vitis vinifera* gene sequences coding for lipoxygenase (LOX)**^1^,** allene oxide synthase (AOS), allene oxide cyclase (AOC), 12-oxo-phytodienoic acid (OPDA) reductase (OPR)**^3^** and jasmonate carboxyl methyltransferase (JMT)**^4^** identified in the NCBI database and used for the synthesis of oligonucleotides for expression analysis.

| **Gene** | **NCBI Reference Sequence** | **ORF (bp)** | **Chromosome location** | **Forward primer (5'-3')** | **Reverse primer (5'-3')** |
| --- | --- | --- | --- | --- | --- |
|
|
| *VvLOX1* | NM_001281094 | 2706 | 6 | TTGATGCTAGGAATGAAGAC | ATGGAGATACTGTATGGAAC |
|
| *VvLOX2* | NM_001303088 | 2940 | 9 | ACACCAACTTCCTCAACG | TCTCTATCCTCCTTATTTCC |
|
| *VvLOX3* | XM_002265469 | 2763 | 1 | CTGCTAAACTAGAGGAGATA | GAAATGCTGTTGGGAATACC |
|
| *VvLOX4* | NM_001281249 | 2580 | 14 | GAACTCAAGTCCAATCCT | GTATGGTATCTTCACAGG |
|
| *VvAOS1* | XM_002281123 | 1452 | 3 | ACTCGCATCTCGCCATCG**^2^** | GTTGAAGTTAGCTTTCCCCTT**^2^** |
|
| *VvAOS3* | XM_002281154 | 1452 | 3 | * | * |
|
| *VvAOS2* | XM_002281190 | 1464 | 3 | ATGGGCAACAGGTTTATG | GAACTCCACCAGCATTAC |
|
| *VvAOS4* | XM_002281165 | 1497 | 3 | ATCAGGAGCATCGTTAAGG**^2^** | CCGTACTGGAATGGAACC**^2^** |
|
| *VvAOS6* | NM_001281219 | 1497 | 3 | * | * |
|
| *VvAOS5* | XM_002283744 | 1563 | 18 | CAGGCTATTTGTGGTGGAG | TTAGGGAGGTTAGATTGATG |
|
| *VvAOS7* | NM_001281082 | 1464 | 12 | AAACTCTCCGCCTCAACC | TCTGTCATCGCCACCTTC |
|
| *VvAOC1* | XM_002278732 | 765 | 1 | AGCCAGCCAACTCACTGG | AGCGGTCACCCTTCCTCTCC |
|
| *VvAOC2* | NM_001280971 | 756 | 14 | GCTTACCTCCGATTGAGCC | ACCTGTCTCCCTTCTTCCC |
|
| *VvOPR1* | NM_001281046 | 1197 | 11 | GTGAAGAAGAGGAAGCTCA | AGGCGACCATAGGATACC |
| *VvJMT1* | XM_003631844 | 1050 | 4 | GGAGGACATAGTGACTGAAA | CTGCTCTCATCGCCTTTGC |
| *VvJMT2* | XM_002281530 | 1137 | 18 | CTGTGAGTGAAGGTGTGTAT | ATCCATTATATGTCCTCCAA |
| *VvJMT3* | XM_002281543 | 1128 | 18 | AGAGAGTTGCTACCAGTG**^4^** | CATCCCAATCTATTTCAAAC^4^ |
| *VvJMT4* | XM_002281552 | 1128 | 18 | * | * |
|

**^1^** *VvLOX4* belongs to the 9-LOX group, whose components attach the hydroperoxide group on the carbon-9 of the fatty acid backbone, whereas *VvLOX1-3*, all having a chloroplast/plastid transit peptide, belong to the 13-LOX group (preferential insertion of the hydroperoxide group on the carbon-13).

**^2^** Due to the remarkable similarity detected among the two pairs of genes *VvAOS1/VvAOS3* and *VvAOS4/VvAOS6* (nucleotide identity of 97 and 98%, respectively), a single set of conserved primers was designed (denoted with asterisks in the table) for each of the two pairs of very similar sequences (referred to as *VvAOS1/3* and *VvAOS4/6* in the text) for expression analyses.

**^3^** Database search allowed to identify eleven distinct *V. vinifera* genes coding for OPR, one of which (*VvOPR1*) was assigned to chromosome 11 and the remaining ten were located in two distinct clusters in the proximal and the distal regions of chromosome 18. *VvOPR1* was the only sequence showing high similarity with the plant *OPR3-like* sequences, involved in jasmonate synthesis. Therefore, only for this sequence specific primers for expression analyses were designed.

**^4^** *VvJMT3* and *VvJMT4* showed a high level of homology among each other. Therefore, a single conserved pair of primers was designed (denoted with asterisks in the table) for studying their expression (denoted as *VvJMT3/4* in the main text), whereas sequence-specific primers were designed for *VvJMT1* and *VvJMT2*.

**Table S3**. *Vitis vinifera* gene sequences involved in salicylate (*NPR1.1*, *NPR1.2* and *EDS1*) [1, 2] or jasmonate (*MYC2*, *JAZ1*, *JAZ2* and *JAZ3*) [3] signalling pathways identified in the NCBI database and used for the synthesis of oligonucleotides for expression analysis.

| **Gene** | **NCBI Reference Sequence** | **ORF (bp)** | **Chromosome location** | **Forward primer (5'-3')** | **Reverse primer (5'-3')** |
| --- | --- | --- | --- | --- | --- |
|
|
| *VvNPR1.1* | XM_002281439 | 1755 | 11 | GCAACGGCACTACAGAGG | AGAGATGTGGATGATGATGAGG |
|
| *VvNPR1.2* | XM_002274009 | 1764 | 10 | GCTCACAAGTCCTGGATAAG | ATGAAGATGAAGATGAAGATAACC |
|
| *VvEDS1* | XM_010664719 | 1791 | 17 | GAATCCTGTTTCTGGGCTGAG | TCTGTAGTTGCTGAATCTTCT |
|
| *VvMYC2* | NM_001281045 | 1827 | 15 | GCCAGCCTTACCAGTGTTG | GCCTCTCTTCTGCCTTTCG |
|
| *VvJAZ1* | XM_002277121 | 807 | 11 | AACCAGTTGCTTGTGAAC | GGAGAGTTGCTTATATTGTATG |
|
| *VvJAZ2* | XM_002272327 | 861 | 9 | ATCGGCAACAACAGAGTC | CAGAAGTGGAGAGGAAGC |
|
| *VvJAZ3* | XM_002284819 | 1167 | 1 | TTCTGCCACTCCTGTTACC | CTCATCACCCTCTCCTTGC |

**References**

1. Chong J, Le Henanff G, Bertsch C, Walter B. Identification, expression analysis and characterization of defence and signalling genes in Vitis vinifera. Plant Physiol Biochem. 2008;46:469-81.
2. Le Henanff G, Heitz T, Mestre P, Mutterer J, Walter B, Chong J. Characterization of Vitis vinifera NPR1 homologs involved in the regulation of pathogenesis-related gene expression. BMC Plant Biol. 2009;9:54.
3. Ismail A, Riemann M, Nick P. The jasmonate pathway mediates salt tolerance in grapevines. J Exp Bot**.** 2012;63:2127-39.

**Table S4**. *Vitis vinifera* gene sequences coding for WRKY**^1^** transcription factors identified in the NCBI database and used for the synthesis of oligonucleotides for expression analysis.

| **Gene** | **NCBI Reference Sequence** | **ORF (bp)** | **Chromosome location** | **Forward primer (5'-3')** | **Reverse primer (5'-3')** |
| --- | --- | --- | --- | --- | --- |
|
|
| *VvWRKY1* | NM_001281289 | 456 | 17 | GGGCAGAAAGCAGTCAAG | CCTCAAGATGTGTTCAAAGTTC |
|
| *VvWRKY2* | NM_001281181 | 1611 | ND | AAGTATGGGCAGAAAGTTGTC | CTGTTGGCTGTGTTGTGG |
|
| *VvWRKY8* | NM_001281019 | 954 | 4 | GAGGTGTCATTAGGCTTCAAC | GACTAAGAACTGCTGGACTA |
|
| *VvWRKY25* | XM_002272468 | 1074 | 8 | GATTCTTCCATTCTGCTCAG | GTTGTTCTTGTTGTTGTTATTG |
|
| *VvWRKY34* | XM_002266152 | 894 | 11 | ATCCTCCGACAACATCTCC | GTAGCCTCGTGGGTATGG |
|
| *VvWRKY45* | XM_010662802 | 1590 | 14 | GAACCAAATCCCAAGAGAAG | CACCTGTAGCCATCATCC |
|
| *VvWRKY51* | XM_002267757 | 1095 | 16 | ATCAGCAATTCAACCTCCAACC | GTGTCCAAGCCTTCTGTTACG |
|

**^1^** *VvWRKY1* and *VvWRKY2* are the only two members of the family which have been functionally characterized [1, 2, 3], whereas the remaining five WRKY genes were chosen because they are either up-regulated by salicylate (*VvWRKY8*, *VvWRKY25* and *VvWRKY51*) [4] or by jasmonate (*VvWRKY34* and *VvWRKY45*) [5].

ND = not determined

**References**

1. Mzid R, Marchive C, Blancard D, Deluc L, Barrieu F, Corio-Costet MF, Drira N, Hamdi S, Lauvergeat V. Overexpression of *VvWRKY2* in tobacco enhances broad resistance to necrotrophic fungal pathogens. Physiol Plantarum. 2007;131:434–47.
2. [Guillaumie S](http://www.ncbi.nlm.nih.gov/pubmed/?term=Guillaumie S%5BAuthor%5D&cauthor=true&cauthor_uid=19902151), [Mzid R](http://www.ncbi.nlm.nih.gov/pubmed/?term=Mzid R%5BAuthor%5D&cauthor=true&cauthor_uid=19902151), [Méchin V](http://www.ncbi.nlm.nih.gov/pubmed/?term=Méchin V%5BAuthor%5D&cauthor=true&cauthor_uid=19902151), [Léon C](http://www.ncbi.nlm.nih.gov/pubmed/?term=Léon C%5BAuthor%5D&cauthor=true&cauthor_uid=19902151), [Hichri I](http://www.ncbi.nlm.nih.gov/pubmed/?term=Hichri I%5BAuthor%5D&cauthor=true&cauthor_uid=19902151), [Destrac-Irvine A](http://www.ncbi.nlm.nih.gov/pubmed/?term=Destrac-Irvine A%5BAuthor%5D&cauthor=true&cauthor_uid=19902151), [Trossat-Magnin C](http://www.ncbi.nlm.nih.gov/pubmed/?term=Trossat-Magnin C%5BAuthor%5D&cauthor=true&cauthor_uid=19902151), [Delrot S](http://www.ncbi.nlm.nih.gov/pubmed/?term=Delrot S%5BAuthor%5D&cauthor=true&cauthor_uid=19902151), [Lauvergeat V](http://www.ncbi.nlm.nih.gov/pubmed/?term=Lauvergeat V%5BAuthor%5D&cauthor=true&cauthor_uid=19902151). The grapevine transcription factor WRKY2 influences the lignin pathway and xylem development in tobacco. Plant Mol Biol. 2010;72:215-34.
3. [Marchive C](http://www.ncbi.nlm.nih.gov/pubmed/?term=Marchive C%5BAuthor%5D&cauthor=true&cauthor_uid=23342101), [Léon C](http://www.ncbi.nlm.nih.gov/pubmed/?term=Léon C%5BAuthor%5D&cauthor=true&cauthor_uid=23342101), [Kappel C](http://www.ncbi.nlm.nih.gov/pubmed/?term=Kappel C%5BAuthor%5D&cauthor=true&cauthor_uid=23342101), [Coutos-Thévenot P](http://www.ncbi.nlm.nih.gov/pubmed/?term=Coutos-Thévenot P%5BAuthor%5D&cauthor=true&cauthor_uid=23342101), [Corio-Costet MF](http://www.ncbi.nlm.nih.gov/pubmed/?term=Corio-Costet MF%5BAuthor%5D&cauthor=true&cauthor_uid=23342101), [Delrot S](http://www.ncbi.nlm.nih.gov/pubmed/?term=Delrot S%5BAuthor%5D&cauthor=true&cauthor_uid=23342101), [Lauvergeat V](http://www.ncbi.nlm.nih.gov/pubmed/?term=Lauvergeat V%5BAuthor%5D&cauthor=true&cauthor_uid=23342101). Over-expression of *VvWRKY1* in grapevines induces expression of jasmonic acid pathway-related genes and confers higher tolerance to the downy mildew. [PLoS ONE](http://www.ncbi.nlm.nih.gov/pubmed/23342101). 2013;8:e54185
4. Wang M, Vannozzi A, Wang G, Liang YH, Tornielli GB, Zenoni S, Cavallini E, Pezzotti M, Cheng ZM. Genome and transcriptome analysis of the grapevine (Vitis vinifera L.) WRKY gene family. Hortic Res. 2014;1:16.
5. Almagro L, Carbonell-Bejerano P, Belchí-Navarro S, Bru R, Martínez-Zapater JM, [Lijavetzky D](http://www.ncbi.nlm.nih.gov/pubmed/?term=Lijavetzky D%5BAuthor%5D&cauthor=true&cauthor_uid=25314001), [Pedreño MA](http://www.ncbi.nlm.nih.gov/pubmed/?term=Pedreño MA%5BAuthor%5D&cauthor=true&cauthor_uid=25314001). Dissecting the transcriptional response to elicitors in *Vitis vinifera* cells**.** PLoS ONE. 2014;9:e109777.

**Table S5**. *Vitis vinifera* gene sequences coding for pathogenesis-related proteins (PRP)**^1^** identified in the NCBI database and used for the synthesis of oligonucleotides for expression analysis.

| **Gene** | **NCBI Reference Sequence** | **ORF (bp)** | **Chromosome location** | **Forward primer (5'-3')** | **Reverse primer (5'-3')** |
| --- | --- | --- | --- | --- | --- |
|
|
| *VvPR1.1* | XM_002273752 | 483 | 3 | TATGACTACAACTCCAACTC**^2^** | ACGAACCACCCTCCATTG**^2^** |
|
| *VvPR1.2* | XM_002273380 | 483 | 3 | * | * |
|
| *VvPR2* | NM_001281224 | 1038 | 8 | AACTCCAATTTGATTCAG | CCAGTGTTTCTCATATTC |
|
| *VvBGL2* | NM_001280967 | 1080 | 5 | GAGCCATAGAGACTTACC | TTTCAGTAGACACATCCC |
|
| *VvCHIT1a* | NM_001280962 | 945 | 3 | GCAGTCATATCATTCAAG | GGACCTGAGGAACTACCC |
|
| *VvCHIT1b* | NM_001281121 | 978 | 4 | GAGTTCAGACACATCAGC | ACTGCCATAGCCTATTCCC |
|
| *VvCHIT4C* | NM_001281244 | 804 | 5 | GCCTTGTGGTATTGGATGAC | GCATTGACAGCAGCAGTG |
|
| *VvPR4* | XM_002264684 | 432 | 14 | CGCCTACTGCTCCACTTG | GTCCACTATTCTCACTGTTGC |
|
| *VvTHAU2* | NM_001281132 | 669 | 2 | GGCGGACGGAGACTTGAC | ATTCAGCGAGGGTGTTTGG |
|
| *VvOsm* | XM_002282952 | 678 | 2 | GTGCCCTGATGCTTATAG | ATACCATTGCCTCCTAATAG |
|
| *VvPIN* | XM_002284418 | 216 | 5 | GAATCCTCATATCACTACTG | GGGACACTGATTACGATGCC |
|
| *VvCHITIII* | NM_001281119 | 906 | 16 | TGGCAACACCAATAACCT | TACTTTGACCACAGCATC |
|
| *VvPR10.1* | XM_002274749 | 480 | 5 | TAAGGGTGGCAAAGAGGA^2^ | ACCACAGGCAATTTTACTTA^2^ |
|
| *VvPR10.3* | NM_001281027 | 480 | 5 | * | * |
| *VvPR10.2* | XM_002274206 | 477 | 5 | GCGCTGAGGTCTGTGAAG | TGACTGATGCTATGAAGG |

**^1^** The above listed sequences can be assigned to eight of the 17 known families of PRP [1], given in parentheses as follows: *VvPR1.1* and *VvPR 1.2* (PR-1); *VvPR2* and *VvBGL2* (PR-2); *VvCHIT1a*, *VvCHIT1b,* and *VvCHIT4C* (PR-3); *VvPR4* (PR-4); *VvTHAU2* and *VvOsm* (PR-5); *VvPIN* (PR-6); *VvCHITIII* (PR-8); *VvPR10.1*, *VvPR10.2* and *VvPR 10.3* (PR-10). Six out of the 15 genes were selected on the basis of their overexpression following exogenous hormone treatments: *VvPR1.1*, *VvPR1.2,* and *VvCHITIII* are induced specifically by salicylate [2, 3, 4], whereas *VvCHIT1b*, *VvPR4* and *VvPIN* by jasmonate [3, 4, 5]. The remaining nine PR genes were selected on the basis of their up-regulation in grapevine in response to different pathogens [2, 6, 7, 8].

**^2^** As sequence similarity was remarkably high among *VvPR1.1* and *VvPR1.2* genes, and among *VvPR10.1* and *VvPR10.3* as well, a single set of conserved primers was designed (denoted with asterisks in the table) for each of the two above gene pairs (referred to as *VvPR1.1/1.2* and *VvPR10.1/10.3*, respectively, in the main text).

**References**

# [**van Loon LC**](http://www.ncbi.nlm.nih.gov/pubmed/?term=van Loon LC%5BAuthor%5D&cauthor=true&cauthor_uid=16602946), [**Rep M**](http://www.ncbi.nlm.nih.gov/pubmed/?term=Rep M%5BAuthor%5D&cauthor=true&cauthor_uid=16602946), [**Pieterse CM**](http://www.ncbi.nlm.nih.gov/pubmed/?term=Pieterse CM%5BAuthor%5D&cauthor=true&cauthor_uid=16602946). Significance of inducible defence-related proteins in infected plants. **Annu Rev Phytopathol.** 2006;44:135-62.

# Busam G, Kassemeyer HH, Matern U. Differential expression of chitinases in *Vitis vinifera* L. responding to systemic acquired resistance activators or fungal challenge. Plant Physiol. 1997;115:1029-38.

1. Hamiduzzaman MM, Jakab G, Barnavon L, Neuhaus JM, Mauch-Mani B. β-amino butyric acid induced resistance against downy mildew in grapevine acts through the potentiation of callose formation and JA signalling. Mol Plant Microbe Interact. 2005;18:819–29.
2. Chong J, Le Henanff G, Bertsch C, Walter B. Identification, expression analysis and characterization of defence and signalling genes in Vitis vinifera. Plant Physiol Biochem. 2008;46:469-81.
3. Belhadj A, Saigne C, Telef N, Cluzet S, Bouscaut J, Corio-Costet MF, Merillon JM. Methyl jasmonate induces defence responses in grapevine and triggers protection against Erysiphe necator. J Agr Food Chem. 2006;54:9119-25.

# Jacobs AK, Dry IB, Robinson SP. Induction of different pathogenesis-related cDNAs in grapevine infected with powdery mildew and treatment with ethephon. Plant Pathol. 1999;48:325–36.

1. [Kortekamp A](http://www.ncbi.nlm.nih.gov/pubmed/?term=Kortekamp A%5BAuthor%5D&cauthor=true&cauthor_uid=16531058). Expression analysis of defence-related genes in grapevine leaves after inoculation with a host and a non-host pathogen. Plant Physiol Biochem. 2006;44:58-67.
2. Fung RW, Gonzalo M, Fekete C, Kovacs LG, He Y, Marsh E, McIntyre LM, Schachtman DP, Qiu W. Powdery mildew induces defence-oriented reprogramming of the transcriptome in a susceptible but not in a resistant grapevine. Plant Physiol. 2008;146:236–49.

**Table S6.** *Vitis vinifera* gene sequences coding for stilbene synthase (STS)**^1^** and for MYB transcription factors specifically interacting with the *STS* promoter [1], identified in the NCBI database and used for the synthesis of oligonucleotides for expression analysis.

| **Gene** | **NCBI Reference Sequence** | **ORF (bp)** | **Chromosome location** | **Forward primer (5'-3')** | **Reverse primer (5'-3')** |
| --- | --- | --- | --- | --- | --- |
|
|
| *VvSTS1* | XM_002272093 | 1179 | 10 | AATCACTCAAGGAAGAAAG | TTCACTCAATTTGTAGCC |
|
| *VvSTS2* | XM_002271335 | 1179 | 10 | * | * |
|
| *VvSTS3* | XM_010664197 | 1179 | 16 | AGAGAATGGTCCCTTTAACG | AACAATGACTCAATTACAATC |
|
| *VvSTS4* | XM_003634015 | 1179 | 16 | * | * |
|
| *VvSTS5* | XM_002262908 | 1179 | 16 | GCATTCCTACAGTTACAAATTAAG | CAATGACTCAAGTACAAATC |
|
| *VvSTS6* | XM_003634018 | 1179 | 16 | * | * |
|
| *VvSTS7* | XM_002263686 | 1179 | 16 | AGGTGAAGGATTGGATTGG | TTACATTAAGACATTGAAGGGT |
|
| *VvSTS8* | XM_002263845 | 1179 | 16 | * | * |
|
| *VvSTS9* | XM_010664202 | 1179 | 16 | GCATTCCTATGGTGACAAATTAA | TAGTTTCGGAGATAAATACCTTA |
|
| *VvSTS10* | XM_003634020 | 1179 | 16 | * | * |
|
| *VvSTS11* | XM_002268806 | 1179 | 16 | * | * |
|
| *VvSTS12* | XM_002263771 | 1179 | 16 | ACAGGTGAAGGATTGGATTGG | CTTAATTTGAAACCGTAGGAATGC |
|
| *VvSTS13* | XM_003634017 | 1179 | 16 | GGGTATTATTTGGTTTTGGG | AAAGACCATTCTCCCTTATT |
|
| *VvSTS14* | XM_003634014 | 1179 | 16 | ATTCCTACGGTTACAAATTAAGTG | CAAAGAAAGTCTAACAATGACTTG |
|
| *VvSTS15* | XM_002278349 | 1179 | 16 | GCATTCCTATGGTTTCTAATTGAG | ACACTATATCCACCAACAATCAC |
|
| *VvSTS16* | XM_002278318 | 1179 | 16 | TTACAGAGGAGGTGCTAC | GCGATAACAGAATGACAA |
|
| *VvSTS17* | XM_002268720 | 1179 | 16 | * | * |
|
| *VvSTS18* | XM_002269350 | 1179 | 16 | * | * |
|
| *VvSTS19* | XM_002278447 | 1179 | 16 | GATGAGATGAGAAGGAAATCAT | CGATGGTCAAGCCTGGTC |
|
| *VvSTS20* | XM_002264419 | 1179 | 16 | GGACCAGGCTTAACCATCG | GACTCCAATTTGATACCGTAGAAC |
|
| *VvSTS21* | XM_003634016 | 1179 | 16 | TGCCACGGGTACAAATTGAG | GAAGCCCTCCAGCAATCAGT |
|
| *VvSTS22* | XM_003634022 | 1179 | 16 | CCAGGCTTGACCATTGAGACC | AGTCCTCCAAGAACGATAAATAAC |
|
| *VvSTS23* | XM_003634023 | 1179 | 16 | * | * |
|
| *VvSTS24* | XM_003634027 | 1179 | 16 | * | * |
|
| *VvSTS25* | XM_003634024 | 1179 | 16 | TTATGGACGAGATGAGAAAG | AGGAATACTGTGGAGGAC |
|
| *VvSTS26* | XM_003634028 | 1179 | 16 | * | * |
|
| *VvSTS27* | XM_003634019 | 1179 | 16 | GGTGTCTTGTTTGGCTTTG | ACATGACTCATGAATAAGTTT |
|
| *VvSTS28* | XM_003634025 | 1179 | 16 | * | * |
|
| *VvSTS29* | XM_003634021 | 1179 | 16 | * | * |
|
| *VvSTS30* | XM_003634026 | 1179 | 16 | * | * |
|
| *VvSTS31* | XM_002264953 | 1179 | 16 | GGGTGTTTTGTTTGGCTTTG | ATGGTGGGAACTTGGACTCTC |
| *VvMYB14* | NM_001281203 | 819 | 7 | GGAGAGCCTTGGGTATGG | GCAGGGTGTAGTAATGTCG |
| *VvMYB15* | XM_002285157 | 762 | 5 | GCACTGGCGTCAAGAATG | GTCCATAGGCGAGTTCCG |

**^1^** In *V. vinifera*, STS are encoded by a complex and multigenic family, with at least 48 members [2, 3]. Of these, only 32 seem to code for full-length and functional proteins, which can be assigned to three phylogenetic groups, denoted with A, B, and C. Those belonging to the B group are reputed to be the most responsive to both biotic and abiotic stress [2]. Of the 31 functional *STS* genes identified in the NCBI database, two (*VvSTS1* and *VvSTS2*), belong to the A group, 19 (*VvSTS3-21*) to the B group, and 10 (*VvSTS22-31*) to the C group.

Due to the complexity of the STS gene family and the high level of conservation among most of their members, it was possible, on the basis of differences detected in the 3' end, to design specific primers for only eight of the thirty one identified STS sequences. For the remaining twenty three genes, nine pairs of conserved primers were designed (denoted with asterisks in the table), each amplifying from two to four very similar sequences (referred to as *VvSTS1/2, VvSTS3/4, VvSTS5/6, VvSTS7/8, VvSTS9/11, VvSTS16/18, VvSTS22/24, VvSTS25/26, and VvSTS27/30* in the main text ).

**References**

1. Höll J, Vannozzi A, Czemmel S, D’Onofrio C, Walker AR, [Rausch T](http://www.ncbi.nlm.nih.gov/pubmed/?term=Rausch T%5BAuthor%5D&cauthor=true&cauthor_uid=24151295), Lucchin M, Boss PK, Dry IB, [Bogs J](http://www.ncbi.nlm.nih.gov/pubmed/?term=Bogs J%5BAuthor%5D&cauthor=true&cauthor_uid=24151295). The R2R3-MYB transcription factors MYB14 and MYB15 regulate stilbene biosynthesis in Vitis vinifera. Plant Cell. 2013;25:4135–49.
2. Vannozzi A, Dry IB, Fasoli M, Zenoni S, Lucchin M. Genome-wide analysis of the grapevine stilbene synthase multigenic family: genomic organization and expression profiles upon biotic and abiotic stresses. BMC Plant Biol. 2012;12:130.
3. [Parage C](http://www.ncbi.nlm.nih.gov/pubmed/?term=Parage C%5BAuthor%5D&cauthor=true&cauthor_uid=22961129), [Tavares R](http://www.ncbi.nlm.nih.gov/pubmed/?term=Tavares R%5BAuthor%5D&cauthor=true&cauthor_uid=22961129), [Réty S](http://www.ncbi.nlm.nih.gov/pubmed/?term=Réty S%5BAuthor%5D&cauthor=true&cauthor_uid=22961129), [Baltenweck-Guyot R](http://www.ncbi.nlm.nih.gov/pubmed/?term=Baltenweck-Guyot R%5BAuthor%5D&cauthor=true&cauthor_uid=22961129), [Poutaraud A](http://www.ncbi.nlm.nih.gov/pubmed/?term=Poutaraud A%5BAuthor%5D&cauthor=true&cauthor_uid=22961129), [Renault L](http://www.ncbi.nlm.nih.gov/pubmed/?term=Renault L%5BAuthor%5D&cauthor=true&cauthor_uid=22961129), Heintz D, Lugan R, Marais GAB, Auborg S, [Hugueney P](http://www.ncbi.nlm.nih.gov/pubmed/?term=Hugueney P%5BAuthor%5D&cauthor=true&cauthor_uid=22961129). Structural, functional, and evolutionary analysis of the unusually large stilbene synthase gene family in grapevine. Plant Physiol**.** 2012;160: 1407-19.

**Table S7**. *Vitis vinifera* gene sequences coding for chalcone synthase (CHS) identified in the NCBI database and used for the synthesis of oligonucleotides for expression analysis.

| **Gene** | **NCBI Reference Sequence** | **ORF** | **Chromosome location** | **Forward primer (5'-3')** | **Reverse primer (5'-3')** |
| --- | --- | --- | --- | --- | --- |
|
|
| *VvCHS1* | XM_002276617 | 1179 | 3 | AGCTGAATGCTAGTAGGC | CCCACTCACCATCTCCCT |
|
| *VvCHS2* | XM_002276910 | 1182 | 14 | GGAAAGGCAGCACAGGTG | CACCCAAGGATGACTACG |
|
| *VvCHS3* | XM_002263983 | 1170 | 5 | AGTCGGCTGAGGAAGGGC | CCAATACCAACAAGAGAAGG |
|
| *VvCHS4* | NM_001281135 | 1365 | 14 | GGCGTTCTGTTTGGATTTG | CATTCCCATCTTCCCTTCAG |
|
| *VvCHS5* | XM_002276606 | 1170 | 15 | GCAATTCTGAACCGATTAG | ATTCTTCTCCTCCCTTCC |

Table S8. *Vitis vinifera* gene sequences coding for candidate reference genes [1, 2, 3, 4] identified in the NCBI database and used for the synthesis of oligonucleotides for the normalization of expression data in qRT-PCR analyses.

| **Gene** | **NCBI Reference Sequence** | **ORF** | **Chromosome location** | **Forward primer (5'-3')** | **Reverse primer (5'-3')** |
| --- | --- | --- | --- | --- | --- |
|
|
| *Vv60SPR* | XM_002270599 | 564 | 5 | GCTCCTCTCGGTCAGAACAC | GCTCTCTCAAACTTCCTTCC |
|
| *VvActin7* | XM_002282480 | 1134 | 4 | GTGCTTAGTGGTGGGTCAAC | TGCTGGAAGGTGCTGAGG |
|
| *VvVATP16* | XM_010649277 | 549 | 3 | CTAATGCGCAGCAGCCTA | TCGGGATGACAAGATAATGC |
|
| *VvUQCC* | XM_002264785 | 858 | 16 | TAGATGGATGAAGGACTTGG | AAGGATAGGCAACTACATTC |
|
| *VvSAND* | XM_002285134 | 1848 | 6 | TCAGTATGTATCTTCGGAGT | CCTTTACCCATTGACAGACC |
|
| *VvGAPDH* | XM_002263109 | 1014 | 17 | CCGTGTTCCTACTGTTGATG | CCTCTGACTCCTCCTTGATG |
|
| *VvEF1-α* | XM_002284888 | 1344 | 6 | GCAGGGTTTGTTAAGATGAT | TCCACGCTCTTGATGACTCC |
|

**References**

1. Gamm M, Heloir MC, Kelloniemi J, Poinssot B, Wendehenne D, Adrian M. Identification of reference genes suitable for qRT-PCR in grapevine and application for the study of the expression of genes involved in pterostilbene synthesis. Mol Genet Genom. 2011;285:273–85.
2. Monteiro F, Sebastiana M, Pais MS, Figueiredo A. Reference gene selection and validation for the early responses to Downy Mildew infection in susceptible and resistant Vitis vinifera cultivars. PLoS ONE. 2013;8:e72998.
3. Reid KE, Olsson N, Schlosser J, Peng F, Lund ST. An optimized grapevine RNA isolation procedure and statistical determination of reference genes for real-time RT-PCR during berry development. BMC Plant Biol. 2006;6:27.
4. Selim M, Legay S, Berkelmann-Lohnertz B, Langen G, Kogel KH, Evers D. Identification of suitable reference genes for real-time RT-PCR normalization in the grapevine-downy mildew pathosystem. Plant Cell Rep. 2012;31:205–16.
